# Supplementary material for: SwabSeq: A sequencing-based platform for high-throughput SARS-CoV-2 testing
Source: Nat Biomed Eng. Author manuscript; Available in PMC 2024 Jan 26. (PMC10810734; doi:10.1038/s41551-021-00754-5)
Supplement: SupplementaryInformation [file NIHMS1957064-supplement-SupplementaryInformation.pdf]

Supplementary Information

Supplemental Results

Improving Limit of Detection Requires Minimizing Sources of Noise. One of the major challenges in running a highly sensitive molecular diagnostic assay is that even a single contaminant or source of noise can decrease the test's analytical sensitivity. In the process of developing SwabSeq, we observed S reads from control samples in which no SARS-CoV-2 RNA was present (**Fig. 1D**). We subsequently refer to these reads as "no template control" (NTC) reads. A key part of SwabSeq optimization has been understanding and minimizing the sources of NTC reads in order to improve the limit of detection (LoD) of the assay. We identified two important sources of NTC reads: molecular contamination and mis-assignment of sequencing reads.

To minimize molecular contamination, we followed protocols and procedures that are commonly used in molecular genetic diagnostic laboratories[1]. To limit molecular contamination, we use a dedicated hood for making dilutions of the synthetic RNA controls and master mix. At the start of each new run, we sterilize the pipettes, dilution solutions, and PCR plates with 10% bleach, followed by UV-light treatment for 15 minutes.

To prevent post-PCR products that are at high concentration from contaminating our pre-PCR processes, we physically separated pre- and post-PCR steps of our protocol into two separate rooms, where any post-PCR plates were never opened within the pre-PCR laboratory space. To further protect from post-PCR contamination, we compared RT-PCR mastermixes with or without Uracil-N-glycosylase (UNG). The presence of UNG in the TaqPath™ 1-Step RT-qPCR Master Mix (Thermofisher Scientific) showed a significant improvement reducing post-PCR contamination of S reads present in the negative patient samples as compared with the Luna One Step RT-PCR Mix (New England Biosciences) (**Supplementary Fig. 16**). The RT-PCR mastermix contains a mix of dTTP and dUTP such that post-PCR amplicons are uracil containing DNA. These post-PCR that are remnants of previously run SwabSeq experiments therefore can be selectively eliminated by UNG. Importantly, this addition does not interfere with downstream sequencing.

A third source of molecular contamination was carryover contamination on the sequencer template line of the Illumina MiSeq[2]. Without a bleach maintenance wash, we found that indices from the previous sequencing run were identified in a subsequent experiment where those indices were not included. While the number of reads for some indices were present at a number of S reads, the presence of carryover contamination affects the sensitivity and specificity of our assay. After an extra maintenance and bleach wash, we substantially reduced the amount of carryover reads present to less than 10 reads **(Supplementary Fig. 17)**.

Another source of NTC reads is mis-assignment of amplicons. Mis-assignment of amplicons occurs when sequencing (and perhaps at a lower rate, oligo synthesis) errors result in an amplicon sequence that originates from the *in vitro* S standard but is mistakenly assigned to the S sequence within a given sample. Only 6 bp distinguishes S from *in vitro* S standard at the beginning of read 1. Sequencing errors can result in *in vitro* S standard reads being misclassified as S reads as error rates appear to be higher in the beginning of the read (**Supplementary Fig. 18A**). If computational error correction of the amplicon reads is too tolerant, these reads may be inadvertently counted to the wrong category. To reduce this source of S read misassignment, we use a more conservative thresholding on edit distance

(**Supplementary Fig. 18B**). Future redesigns or extensions to additional viral amplicons should consider engineering longer regions of sequence diversity here.

An additional source of NTC reads is when S amplicon reads are mis-assigned to the wrong sample based on the indexing strategy. In our assay, individual samples are identified by pairs of index reads (**Fig. 1B**). Mis-assignment of samples to the wrong index could occur if there is contamination of index primer sequences, synthesis errors in the index sequence, sequencing errors in the index sequences, or "index hopping" [3].

We leveraged multiple indexing strategies in our development of SwabSeq, from fully combinatorial indexing (where each possible combination of i5 and i7 indices was used to tag samples in the assay) to unique-dual indexing (UDI) where each sample has distinct and unrelated i7 and i5 indices (**Supplementary Fig. 19**). However, the ability to scale can be limited due to the substantial upfront cost of developing that many unique primers. Fully combinatorial indexing approaches significantly expand the number of unique primer combinations. We have also explored a compromise strategy between fully combinatorial indexing and UDI where sets of indices are only shared between small subsets of samples. Such designs reduce the effect of sample mis-assignment while facilitating scaling to tens of thousands of patient samples (**Supplementary Fig. 19**). With a fully combinatorial indexing (**Supplementary Fig. 19**) we observed that NTC read depth was correlated with the total number of S reads summed across all samples that shared the same i7 sequence (**Supplementary Fig. 20A**). This is consistent with the effect of index hopping from samples with high S viral reads to samples that share the same indices. It is possible to computationally correct for this effect, for example using a linear mixed model (**Supplementary Fig. 20B**).

Finally, the challenges associated with combinatorial and semi-combinatorial indexing strategies can be mitigated by using unique dual indexing (UDI), a known strategy to reduce the number of index-hopped reads by two orders of magnitude[4]. We have observed consistently lower S viral reads for negative control samples with this strategy. It also enables quantification of index mis-assignment by counting reads for index combinations that should not occur in our assay (**Supplementary Fig. 21 A and B**). The number of index hopping events is correlated with the total number of S + S spike reads (**Supplementary Supplementary Fig. 21 C and D**), indicating that hopped reads are more likely to come from wells where the expected index has strong viral signal. We quantify the overall rate of hopping as 1-2% on a MiSeq, and expect this rate may be higher on patterned flow cell instruments.

There are many sources of noise in amplicon-based sequencing, from environmental contamination in the RT-PCR and sequencing steps to misassignment of reads based on computational correction and "index-hopping" on the Illumina flow cells. Preventing and correcting these sources of error considerably improves the limit of detection of the SwabSeq assay.

Extension of SwabSeq to additional amplicons in SARS-Co-V2 and to Influenza A/B. We have designed and tested amplicons as extensions of SwabSeq. The motivation behind these additions are that SARS-CoV-2 continues to evolve and could potentially develop mutations that impact the binding sites of our primer pairs and ultimately decrease the sensitivity of our assay. In addition, the utility of our assay beyond the SARS-CoV-2 pandemic requires additional other respiratory viruses that may be concurrently circulating in the population. We have provided the designs for N1 amplicon and for an N1-diversified Standard design (**see Methods**, **Supplementary Table 1).** Our initial validations show that the N1 primer has a limit of detection of 2000 copies/mL in saliva (**Supplementary Fig. 22A**) demonstrating similar detection sensitivity as our S primer. Additional validations for multiplexing primers are ongoing.

Influenza A/B (**see Methods, SupplementaryTable 1**) represent further extension of our assay beyond SARS-CoV-2. As the clinical presentation of COVID-19 is similar to other respiratory viral pneumonia, distinguishing the different viral etiologies will allow for early, precise and preventative treatment of infected patients. We show that these primers are detectable by sequencing assays (**Supplementary Fig. 22B**).

1. Furtado, L. V. *et al.* The 2013 AMP Clinical Practice Committee consisted of Matthew J. Bankowski, Milena Cankovic, Jennifer Dunlap.
2. Nelson, M. C., Morrison, H. G., Benjamino, J., Grim, S. L. & Graf, J. Analysis, optimization and verification of Illumina-generated 16S rRNA gene amplicon surveys. *PLoS One* **9**, e94249 (2014).
3. Valk, T. van der *et al.* Index hopping on the Illumina HiseqX platform and its consequences for ancient DNA studies. *Molecular Ecology Resources* (2019) doi:10.1111/1755-0998.13009.
4. MacConaill, L. E. *et al.* Unique, dual-indexed sequencing adapters with UMIs effectively eliminate index cross-talk and significantly improve sensitivity of massively parallel sequencing. *BMC Genomics* **19**, 30 (2018).

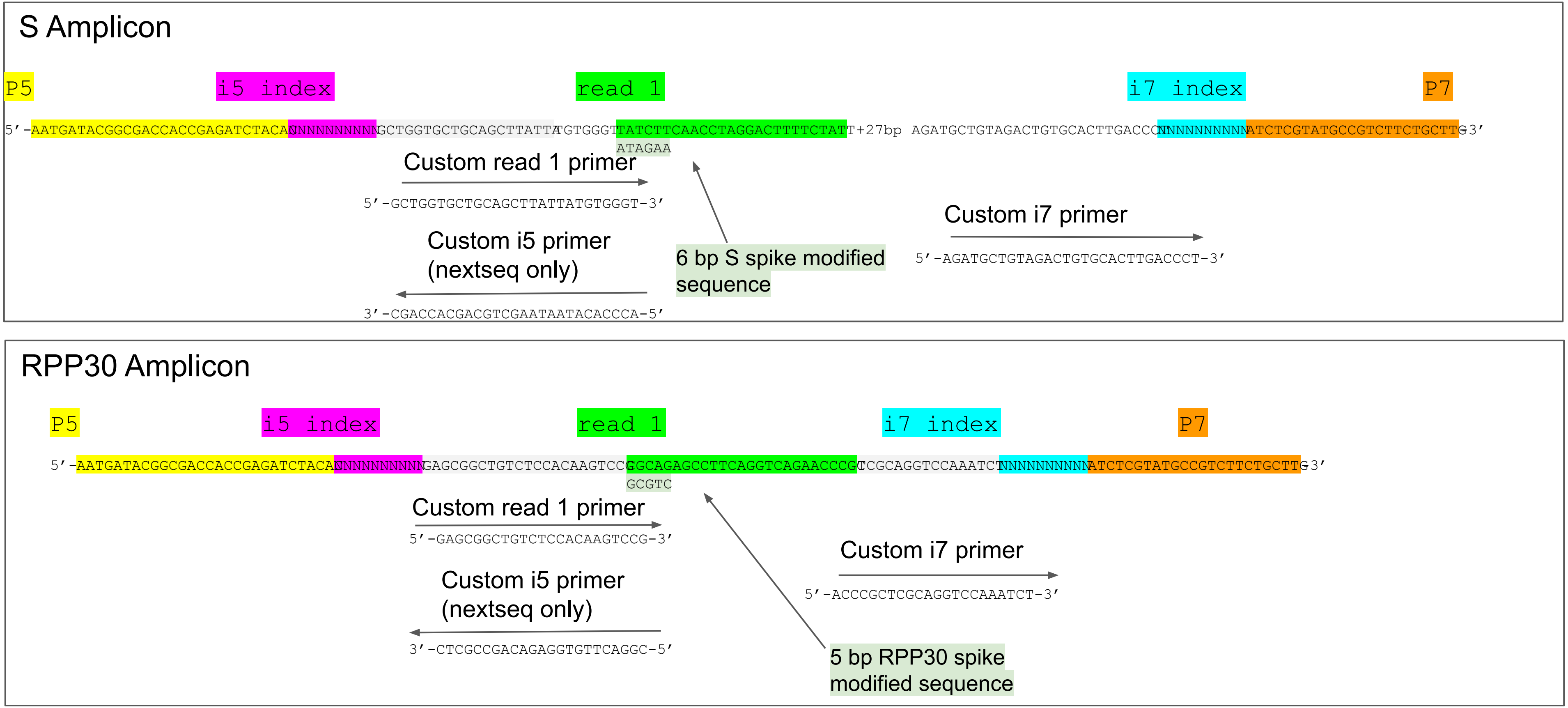

Supplementary Fig. 1. Sequencing library design. The amplicon designs are shown for the S (top) and RPP30 (bottom) amplicons. Amplicons were designed such that the i5 and i7 molecular indexes uniquely identify each sample. SwabSeq was designed to be compatible with all Illumina platforms.

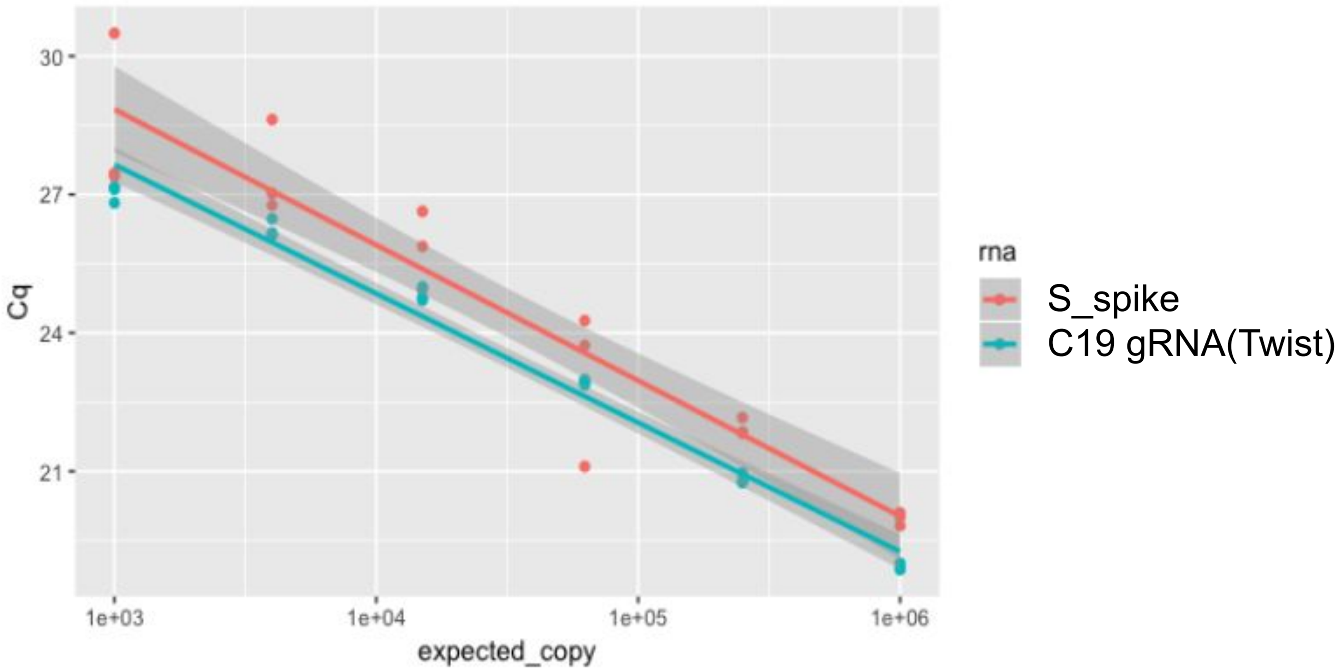

Supplementary Fig. 2. The S primers show equivalent PCR efficiency when amplifying the COVID-19 S gene amplicon and the synthetic *in vitro* S standard. The lines represent a linear regression of Cq on expected copy for each RNA input. Slope of PCR efficiency of the primers with either the S_spike (labeled in red) or the SARS-CoV-2 viral (labeled in green as C19gRNA) input are as follows: S_spike slope = -6.68e-6 and C19gRNA (Twist Control) slope = -6.74e-6. The slopes are expected to equivalent (parallel) if the primers do not show preferential amplification of the S spike RNA versus the C19gRNA. This shows that the S spike and C19gRNA have equivalent amplification efficiencies using the S primer pair. The bands represent 95% confidence intervals for predicted values, are non-overlapping due to different intercepts, and are not relevant for this analysis of slopes.

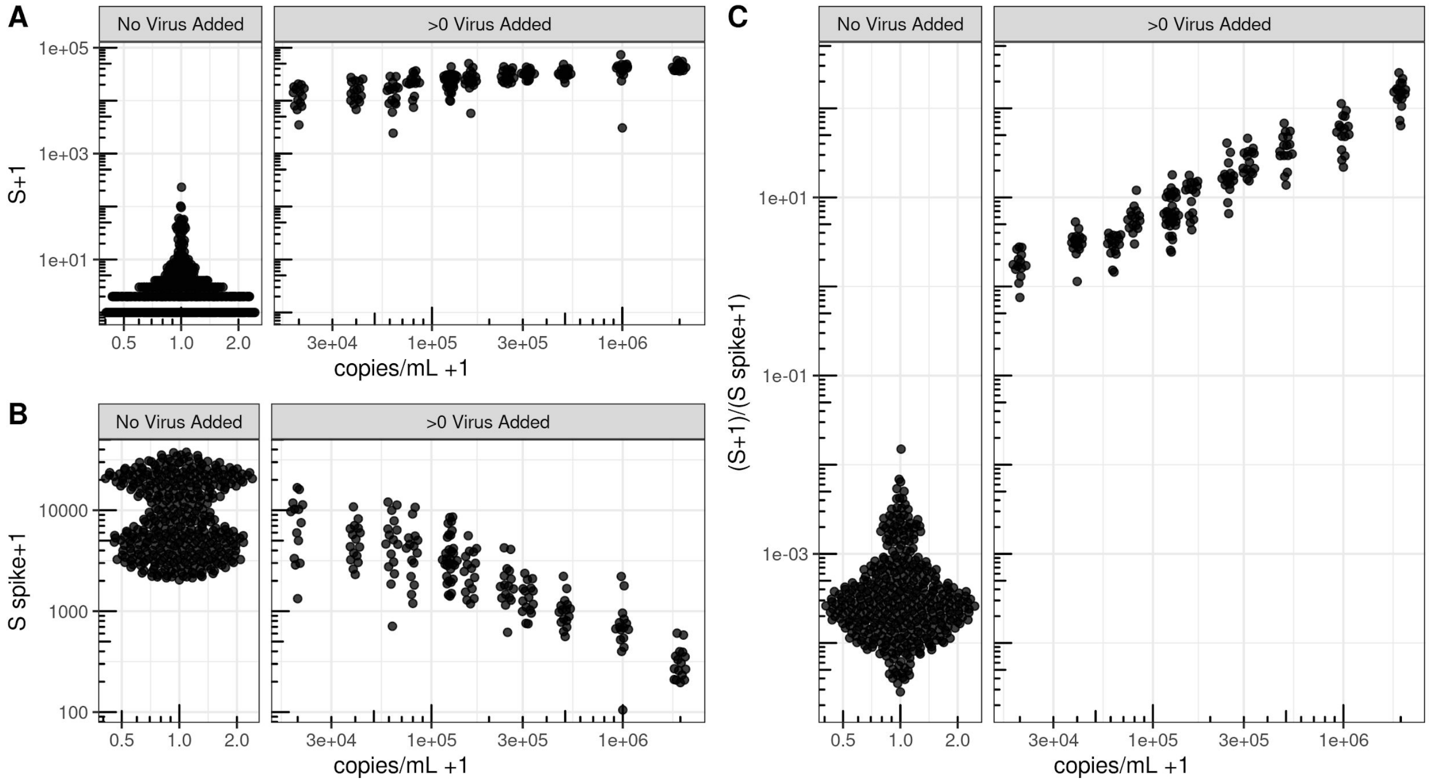

Supplementary Fig. 3. At very high viral concentrations SwabSeq maintains linearity. We include an internal well control, the S Spike, to enable us to call negative samples, even in the presence of heterogeneous sample types and PCR inhibition. (A) As virus concentration increases, we observe increased reads attributed to S and (B) decreased reads attributed to the S Spike. (C) The ratio between the S and S Spike provides an additional level of ratiometric normalization and exhibits linearity up to at least 2 million copies/mL of lysate. Note that ticks on both axes are spaced on a log10 scale.

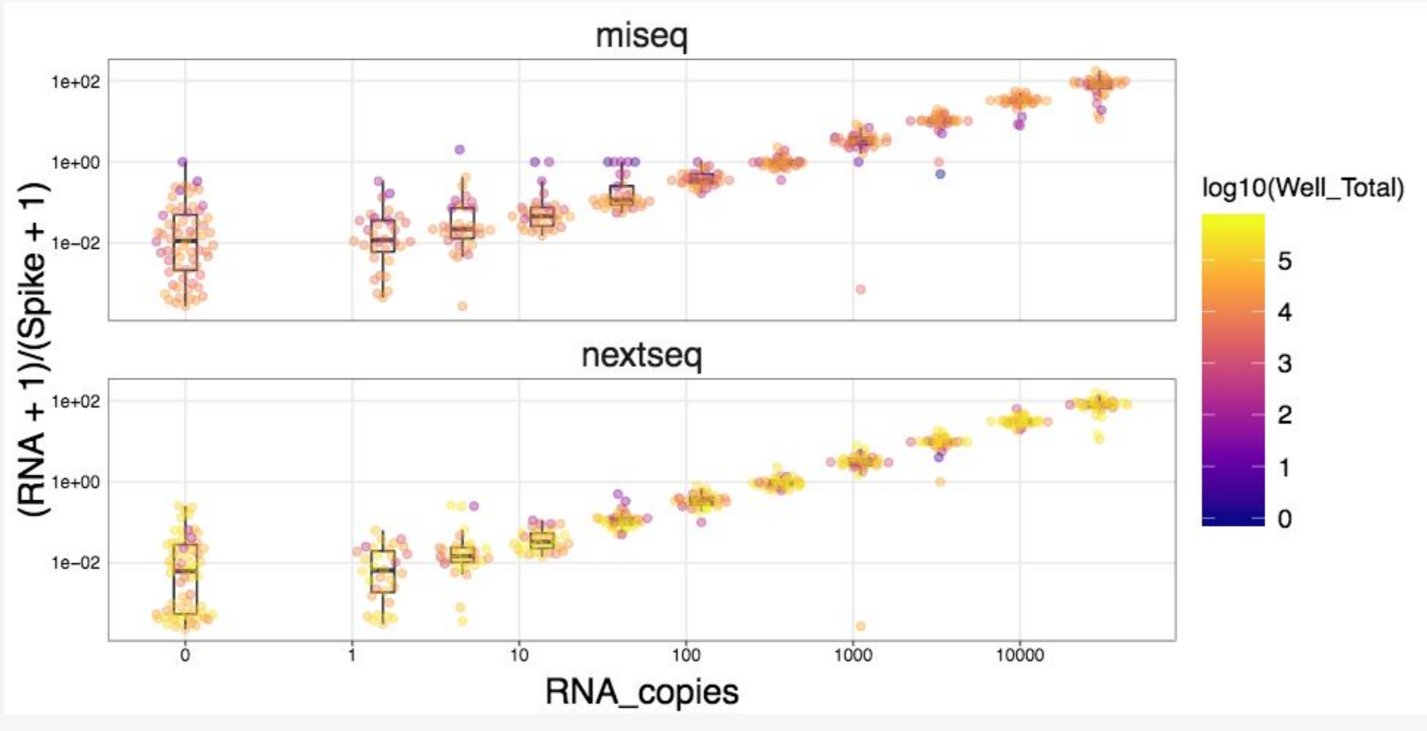

Supplementary Fig. 4. Sequencing is performed on MiSeq or NextSeq Machine with similar sensitivity. Multiplexed libraries run on both MiSeq and NextSeq showed linearity across a wide range of SARS-CoV2 virus copies in a purified RNA background. Data are presented as the ratio of counts with n>=24 replicates per SARS-CoV2 virus copy number. Boxplots show the interquartile range (IQR) and median. Whiskers represent the minimum or maximum of the IQR+1.5*(IQR) and are the default options in R.

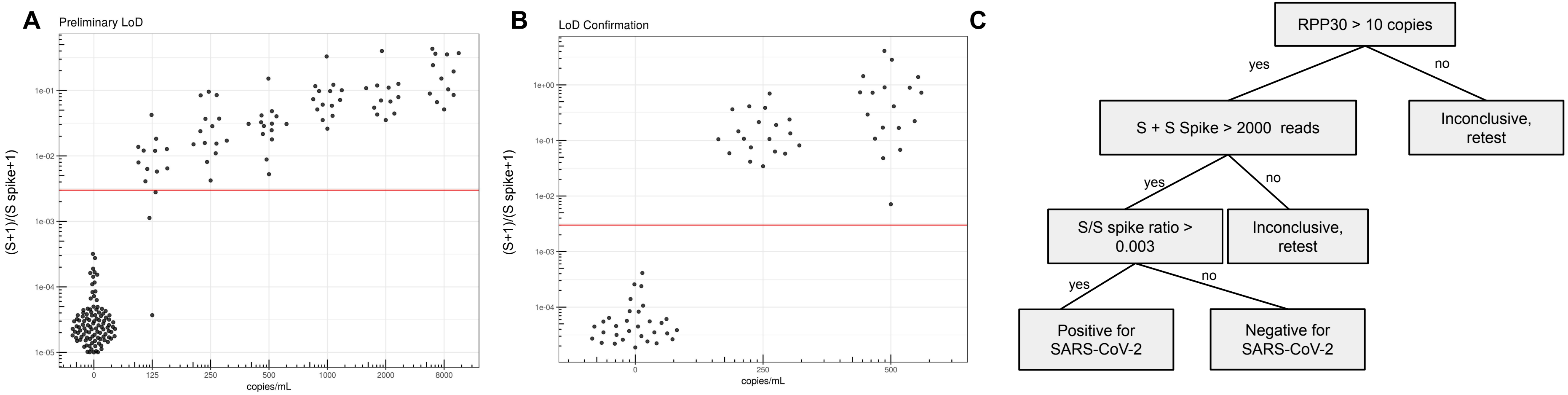

D

		UCLA RT-qPCR		
		**Positive**	**Negative**	
**SwabSeq**	**Positive**	94	0	94
	**Negative**	0	282	282
	**Inconclusive**	0	4	4
	Total	94	286	380
	Positive Percent Agreement			1
	Negative Percent Agreement			98.6%
	Overall Agreement			98.9%

Supplementary Fig. 5. Preliminary and Confirmatory Limit of Detection Data for RNA purified Samples A) Our preliminary LOD data identified a LOD of 250 copies/mL using the NextSeq550 B) Confirmatory studies showed an LOD of 250 copies/mL using the NextSeq550. C) Our result interpretation guidelines for purified RNA. D) Concordance of the 380 clinical samples that were run during our validation process. Concordance is 98.6%, with 100% positive percent agreement (PPA) and 100% negative percent agreement (NPA).

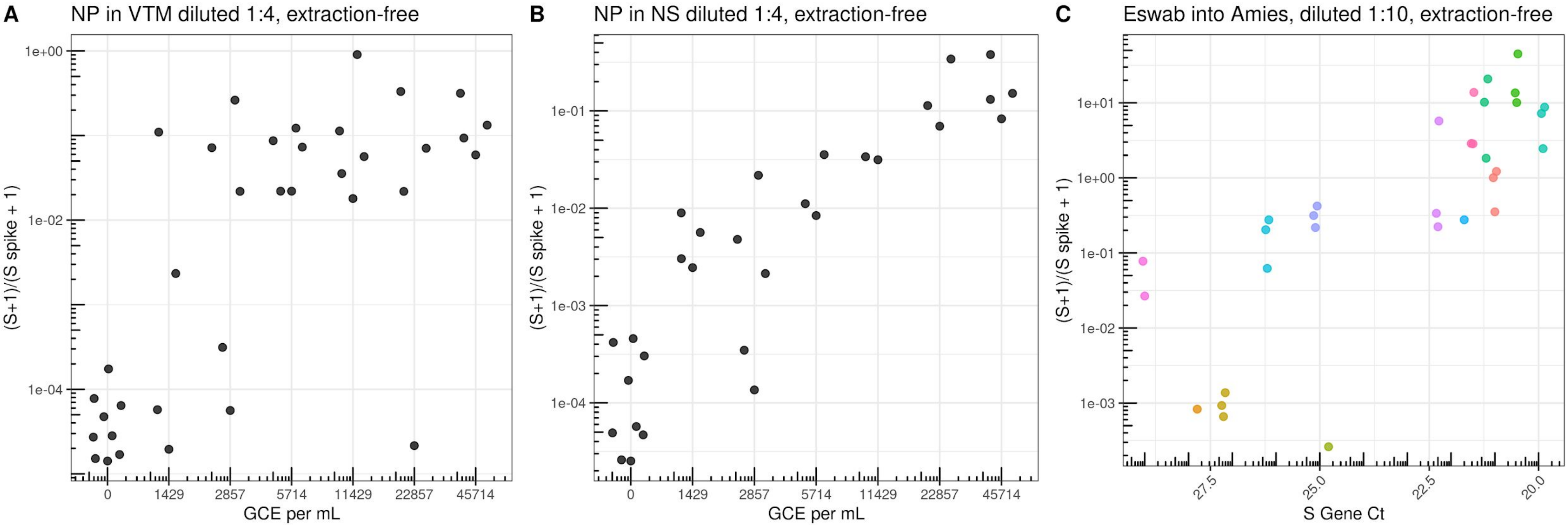

Supplementary Fig. 6. Extraction-Free protocols using traditional collection medias and buffers require dilution to overcome effects of RT and PCR inhibition. A) We tested extraction free protocols for nasopharyngeal swabs that were placed into viral transport media (VTM). We spiked ATCC live inactivated virus at varying concentrations into pooled VTM and then diluted samples 1:4 with water before adding to the RT-PCR reaction. We observed a limit of detection of 5714 copies per mL. B) We also tested nasopharyngeal (NP) swabs that were collected in normal saline (NS), pooled and then spiked with ATCC live inactivated virus at varying concentrations. Contrived samples were diluted 1:4 in water. Here, our early studies show a similar similar limit of detection between 2857 and 5714 copies per mL. C) We tested natural clinical samples that were collected into Amies Buffer (ESwab). Here we compare S gene Ct count (x-axis) from positive samples to the SwabSeq S to S spike ratio (y-axis). Samples were run in triplicate (colors). We observed high concordance for Ct counts of 27 and lower but more variability for Ct counts greater than 27 suggesting that RT and PCR inhibition were affecting our limit of detection. Based on these data we opted only to further explore extraction free protocols into normal saline or tris-EDTA buffer.

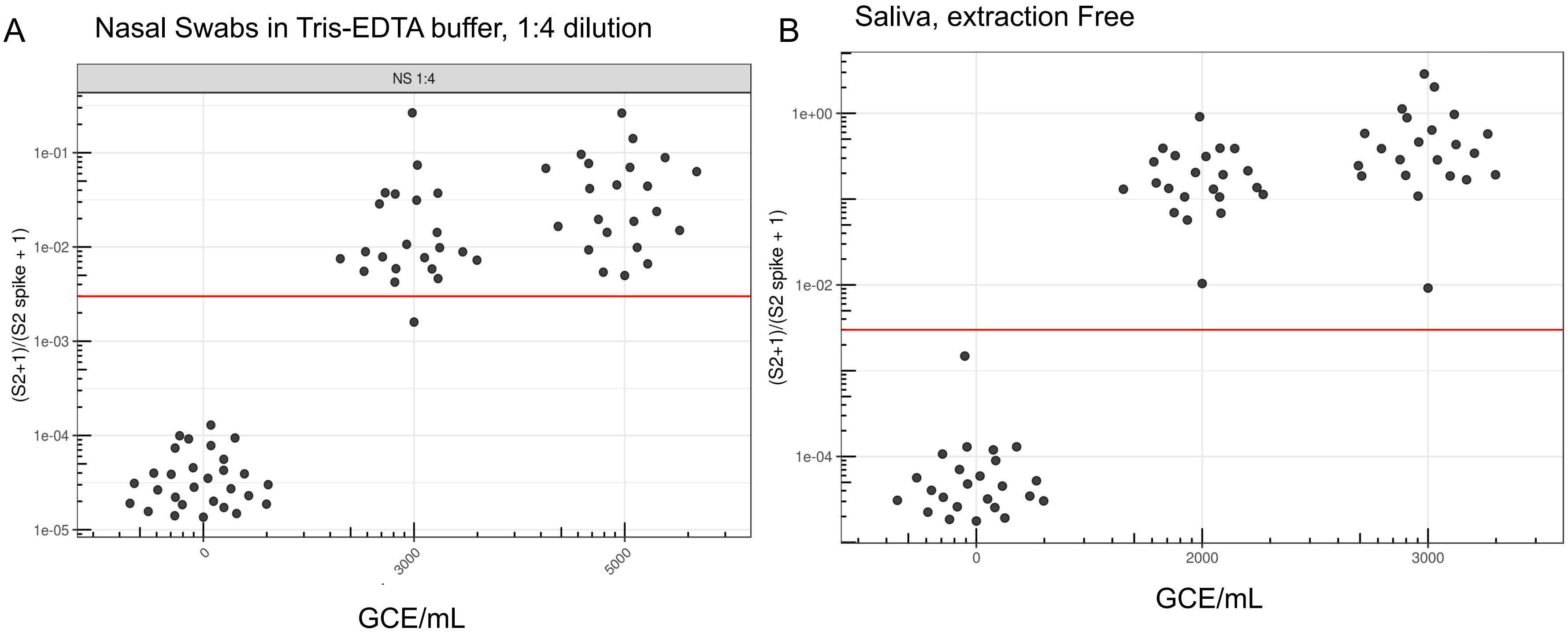

Supplementary Fig. 7. Confirmatory Limit of Detection Data for Extraction Free specimens. A) Our data showed a LOD of 3000 copies/mL for Nasal Swabs samples that were diluted 1:4 in water. These dilutions were performed in replicate at 20 samples per concentration. We tested multiple replicates around the limit of detection. B) Confirmatory studies for extraction-free saliva samples showed an LOD of 2000 copies/mL. Red line indicates the threshold for positivity.

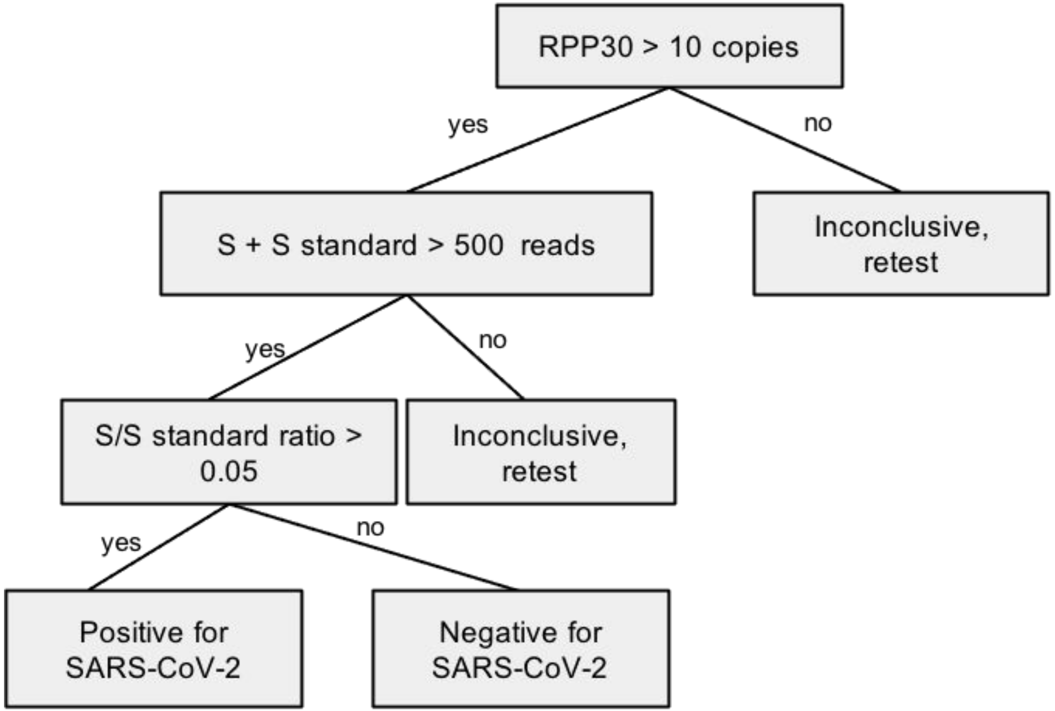

Supplementary Fig. 8. Per sample decision tree for extraction-free samples. Given the slight modifications for our extraction free protocols, we have modified our decision tree to reflect the differences in extraction-free sample types. Our result interpretation guidelines for extraction-free samples relax threshold for S + S spike to 500 reads due to the increased PCR inhibition observed in extraction free sample types. The standard used in our early validations had a slightly lower S/S standard ratio of 0.03 which ultimately in clinical testing had too many false positives. Our current validated test uses the above ratio for extraction free samples.

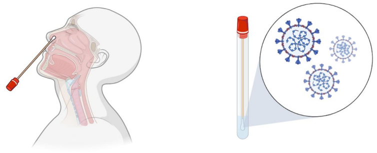

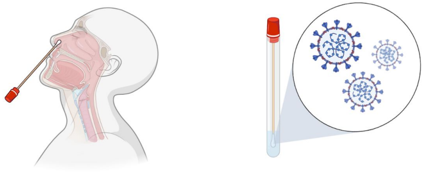

		UCLA RT-qPCR		
		Positive	Negative	
SwabSeq	Positive	80	0	80
	Negative	6	206	212
	Inconclusive	1	5	6
		87	211	298

	Positive Percent Agreement	92.0%
	Negative Percent Agreement	97.6%
	Overall Agreement	96.0%

Supplementary Fig. 9. Comparison of extraction-free NP samples run on SwabSeq to NP Swab samples processed to Clinical pathway using RNA purification and RT-qPCR. Evaluation of extraction free nasal swabs processed into normal saline or Tris-EDTA ph 8.0 that have previously tested positive or negative in the UCLA Clinical Microbiology Laboratory. We have explored the sources of false negatives in our data set. Three of the four false negatives stem from differences in the limit of detection, where we do not always detect samples with Ct > 30.

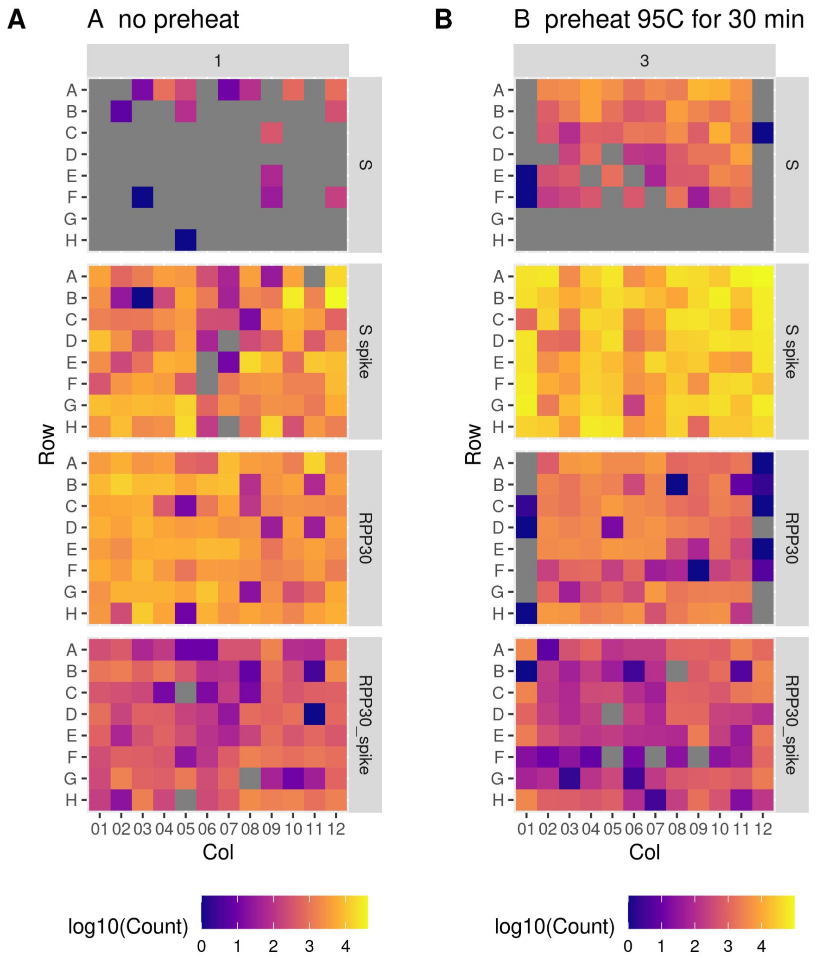

Supplementary Fig. 10. Preheating Saliva to 95C for 30 minutes drastically improves RT-PCR. Detection of viral genome and shows improved robustness in detection of our controls. A) Without preheating, detection of S spike is minimal and there are lower counts for the control amplicons. B) with a 95C preheating step for 30 minutes, we observe robust detection of the S amplicon and synthetic S Spike.

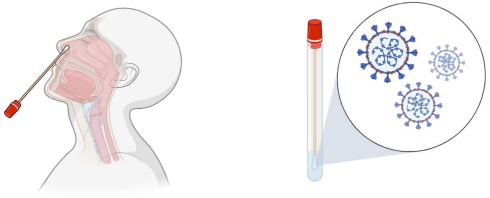

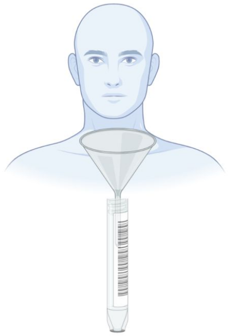

		UCLA RT-qPCR NP Swab		
		Positive	Negative	
SwabSeq Saliva	Positive	74	2	76
	Negative	6	438	444
	Inconclusive	2	15	17
		82	455	537

Positive Percent Agreement	90.2%
Negative Percent Agreement	96.3%
Overall Agreement	95.3%

Supplementary Fig. 11. Comparison of extraction-free saliva samples run on SwabSeq to NP Swab samples processed to Clinical pathway using RNA purification and RT-qPCR. We performed a series of studies to compare the concordance of Saliva and NP swab performed within 2 hours of each other. These collections were obtained in the UCLA ED and UCLA Student Health Center over the course of several months.

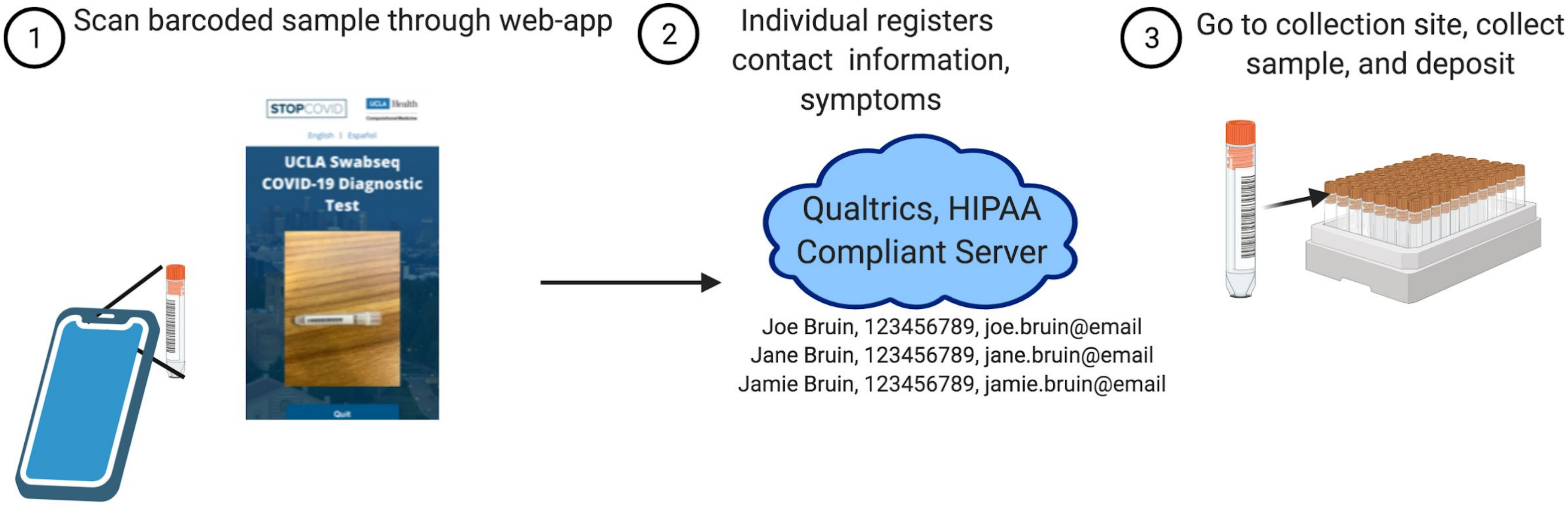

Supplementary Fig. 12. Developing a lightweight sample accessioning to allow for scalable testing into the thousands of samples per day. A major bottleneck is the sample registration to an individual patient. To facilitate the sample accessioning we developed a web-based app for individuals to register their sample tube using a barcode reader and send their identifying information into a secure instance of Qualtrics. In scaled clinical testing in our CLIA laboratory, we used an instance of PreciseQ MDX that allowed organizations to invite cohorts for testing based on their specific needs.

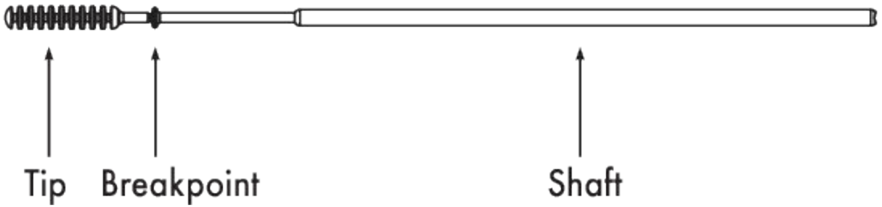

Supplementary Fig. 13. Developing a automation compatible swab. A major bottleneck is the sample processing is the manual process of opening tubes and removing the swab. Not only is this manual but also a source of both cross contamination and biohazardous exposure. To limit this, we designed a 3D printed swab, in conjunction with Applied Medical Company where the breakpoint was engineered to break close to the swab edge such that it would not interfere with our automated pipette machinery.

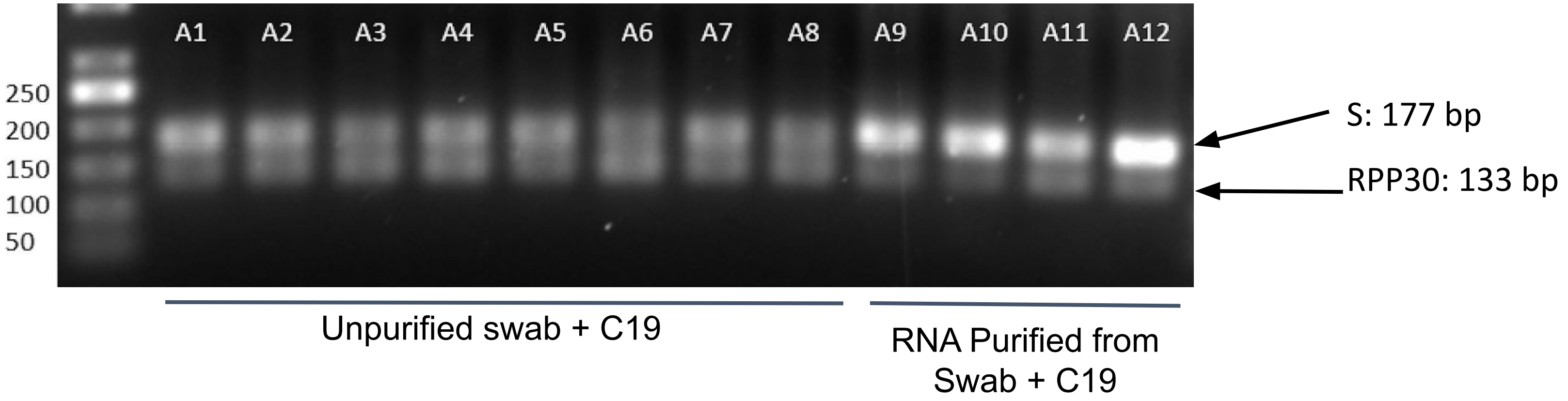

Supplementary Fig. 14. PCR inhibition has significant effect on amplification products. A) 2% Agarose gene was run for a subset of wells from our Rt-PCR reactions. We observe RT-PCR inhibition from swabs in unpurified lysate (A1-A8) (n=8 independent replicates) as compared to purified RNA (A9-A12) (n=4 independent replicates). We observe two bands in this subset of wells representing 2 amplicons for the S or S spike (177bp) and RPP30 (133 bp) primer pairs. An uncropped version of this gel image can be found in **(https://doi.org/10.6084/m9.figshare.14583081.v1)**.

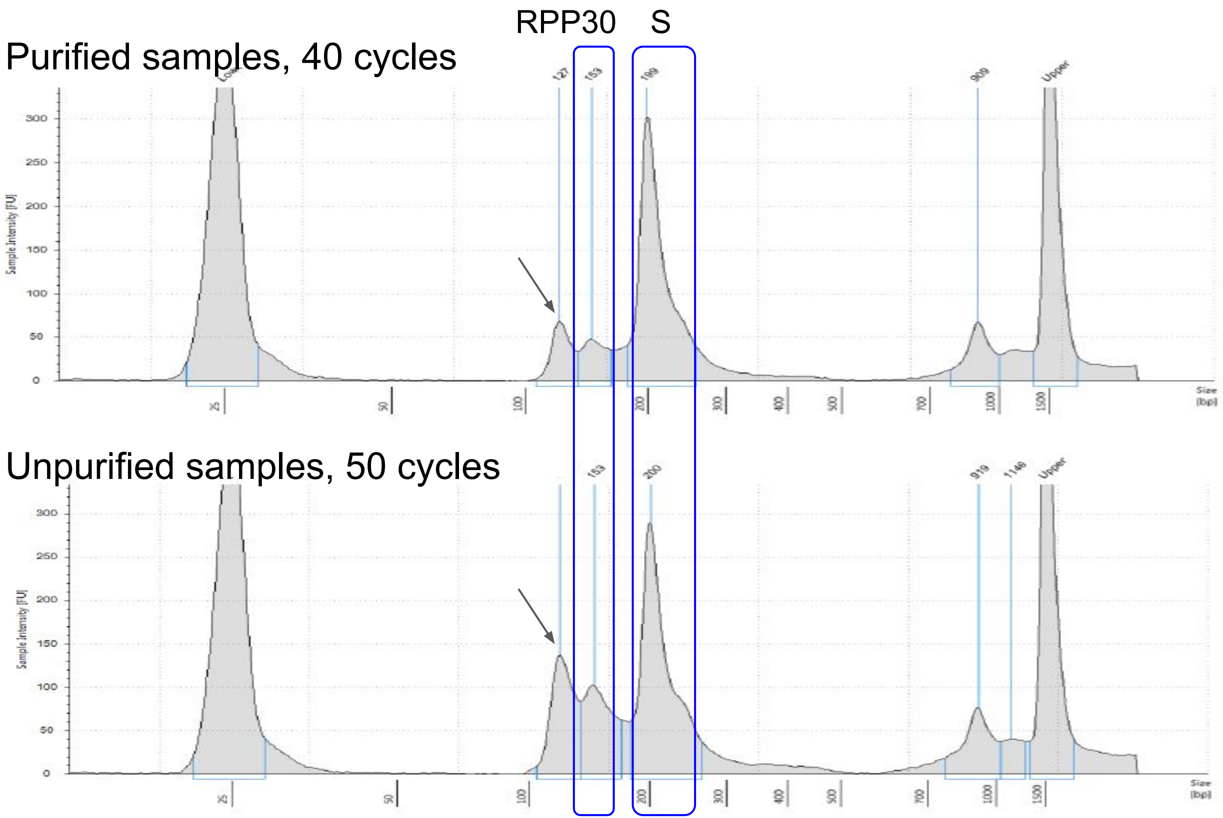

Supplementary Fig. 15. Tapestation Increasing the number of PCR cycles and working with unpurified or inhibitory samples types (eg. Saliva) was seen to increase the size of a nonspecific peak in our library preparation. Representative result from Agilent TapeStation for our purified amplicon libraries. We observe a nonspecific peak slightly above 100bp (arrow) in both library traces, but this peak increases in size with unpurified samples and an increased number of PCR cycles. While we have not confirmed the identity of this peak, we believe this peak may be the result of adapter dimers or unsequenceable PCR artifacts. Importantly, we observe that an increase in the size of this nonspecific peak leads to inaccurate library quantification. Therefore, in order to optimize cluster density on Illumina sequencers, we suggest quantifying the loading concentration of the final library based on the proportion of the desired peaks (RPP30 and S).

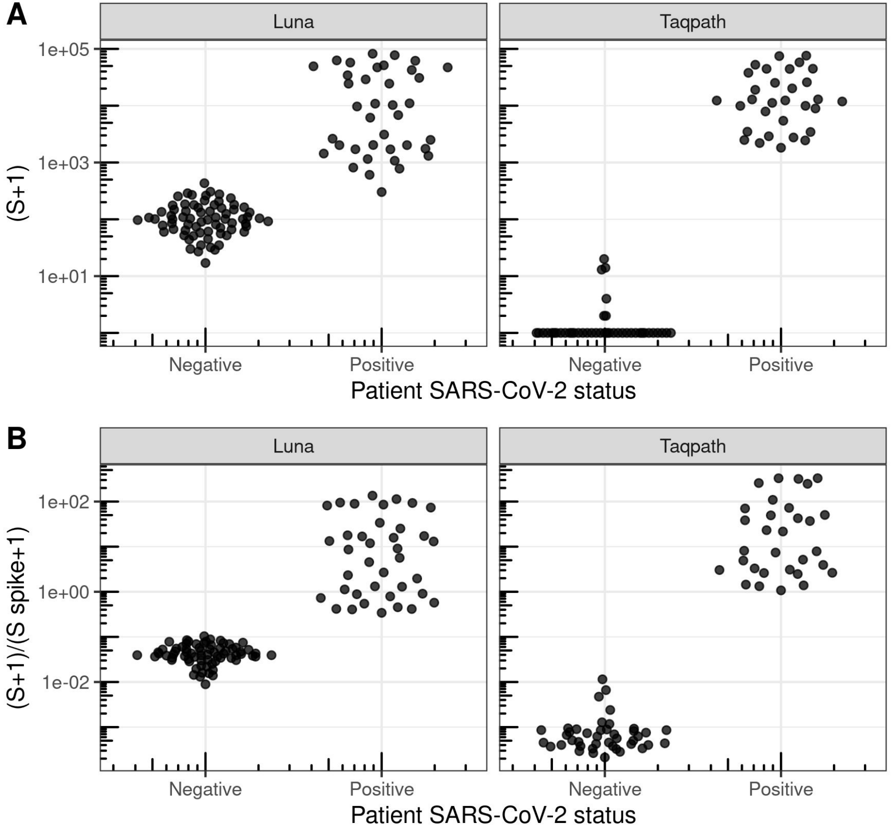

Supplementary Fig. 16. TaqPath decreases the number of S reads in SARS-CoV2-negative samples relative to NEB Luna. We compared Luna One Step RT-PCR Mix (New England Biosciences) to TaqPath™ 1-Step RT-qPCR Master Mix (Thermofisher Scientific). It is likely that the presence of UNG in the TaqPath Mastermix significantly reduced the number of S reads in the SARS-CoV-2-negative samples allowing us to more accurately distinguish SARS-CoV-2-positive and SARS-CoV-2-negative samples.

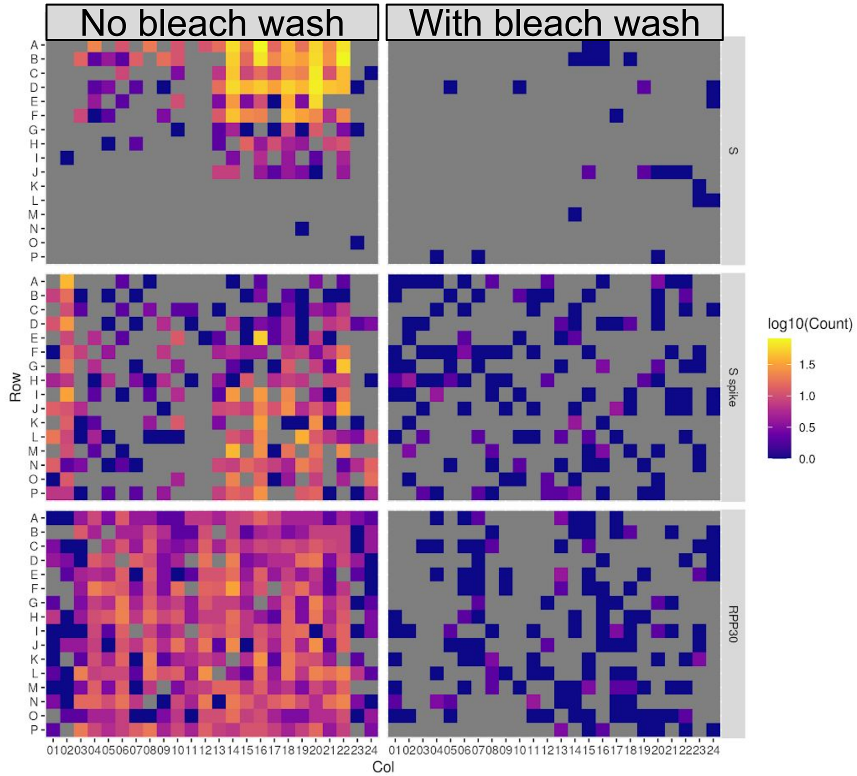

Supplementary Fig. 17. Carryover contamination from template line in a MiSeq contributes to cross contamination. In this experiment we did RT-PCR on four 384-well plates but only pooled three plates. On the left are observed counts of each of the amplicons for each sample for the 384-well plate not included in our run (but for which the indices were used in the previous run). Amplicon reads for indices used in the previous run are present at a low level (0-150 reads). We then performed a bleach wash in addition to regular wash prior to the subsequent run. In this subsequent run, we pooled three different plates and left out the fourth 384 well plate. On the right are observed counts of each of the amplicons for sample indices corresponding to the left-out plate (again, for which the indices were used in the previous run). We observe a remarkable decrease in the amount of carryover contamination, where carryover reads are <10 per sample.

A

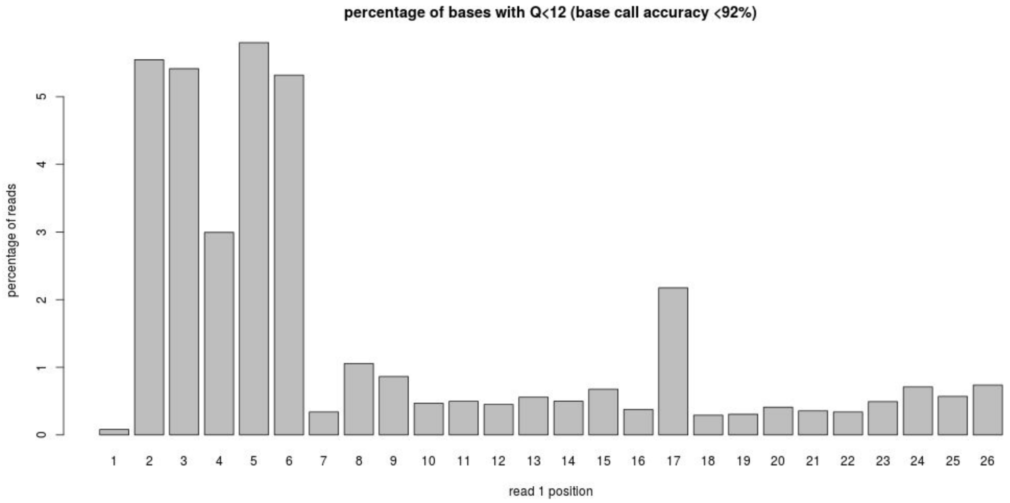

B

Hamming distance from S \ Hamming distance from S spike	0	1	2	3	4	5	6
0	0	0	0	0	0	0	981607
1	0	0	0	0	0	49010	235315
2	0	0	0	0	22578	19024	111529
3	0	0	0	25516	7089	15226	59012
4	0	0	74519	8036	7576	10021	36688
5	0	522655	21671	9469	3292	5824	13217
6	9062990	222210	48991	2270	1337	1669	2140
7	0	928836	19700	2439	277	253	348

Supplementary Fig. 18. Sequencing errors in amplicon read and potential amplicon mis-assignment. In experiment v18 we loaded less PhiX than usual (11%) and the overall quality of read1 was lower. Trends noticed here persist in other runs but this run more clearly highlights issues that can occur due to sequencing errors and overly tolerant error-correction. A) The percentage of reads with base quality scores less than 12 for each position in read 1. Note that the first 6 bases of read1 distinguish S from S spike and have the highest percentage of low quality base calls. B) The hamming distance between each read1 sequence and either the expected S sequence (rows) or S spike sequence (columns), In yellow are perfect match and edit distance 1 sequences that can be clearly identified as S or S spike. In red are sequences with errors that may be mis-assigned (S spike assigned as S is most problematic for this assay.)

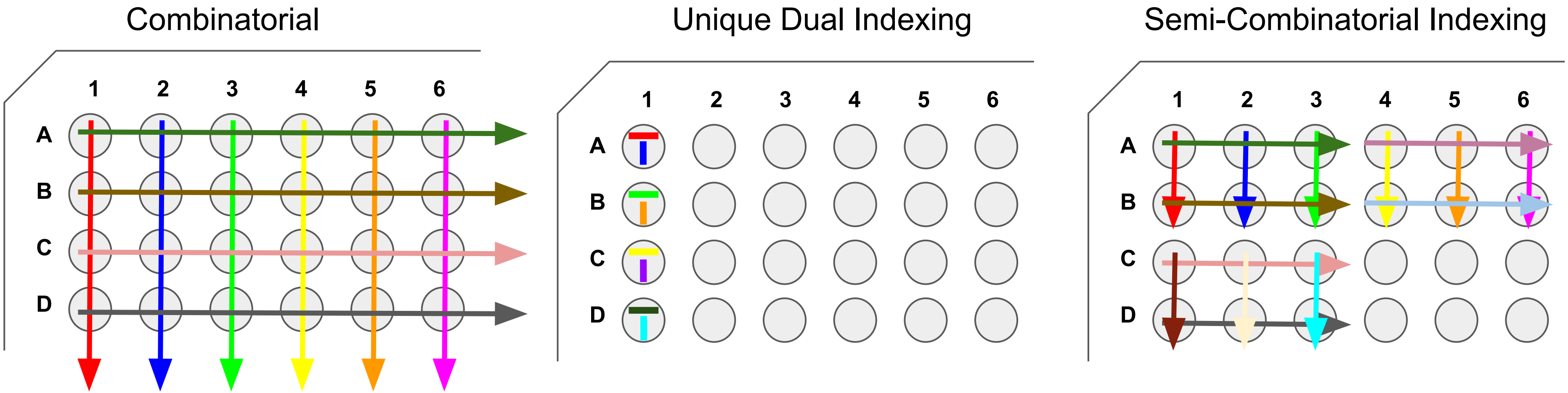

Supplementary Fig. 19. Visualization of different indexing strategies. Here i5 indices are depicted as horizontal lines, i7 indices are depicted as vertical lines, and colors represent unique indices. In combinatorial (or fully-combinatorial) indexing, the i5 and i7 indices are combined to make unique combinations, but each i5 and i7 index may be used multiple times within a plate, and all possible i5 and i7 . For unique dual indexing, each i5 and i7 index are only used 1 time per plate. This requires many more oligos to be synthesized. For Semi-Combinatorial indexing, the combinations used are more limited, such that indices are only repeated for a subset of wells and many possible combinations are not used. In practice (not depicted here), we've used a design where the i7 index is unique but the i5 index can be repeated up to four times across a 384-well plate. For the majority of our SwabSeq development, we used either semi-combinatorial indexing (384x96) that allowed for 1536 combinations or samples to be run or unique dual indexing (384 UDI)

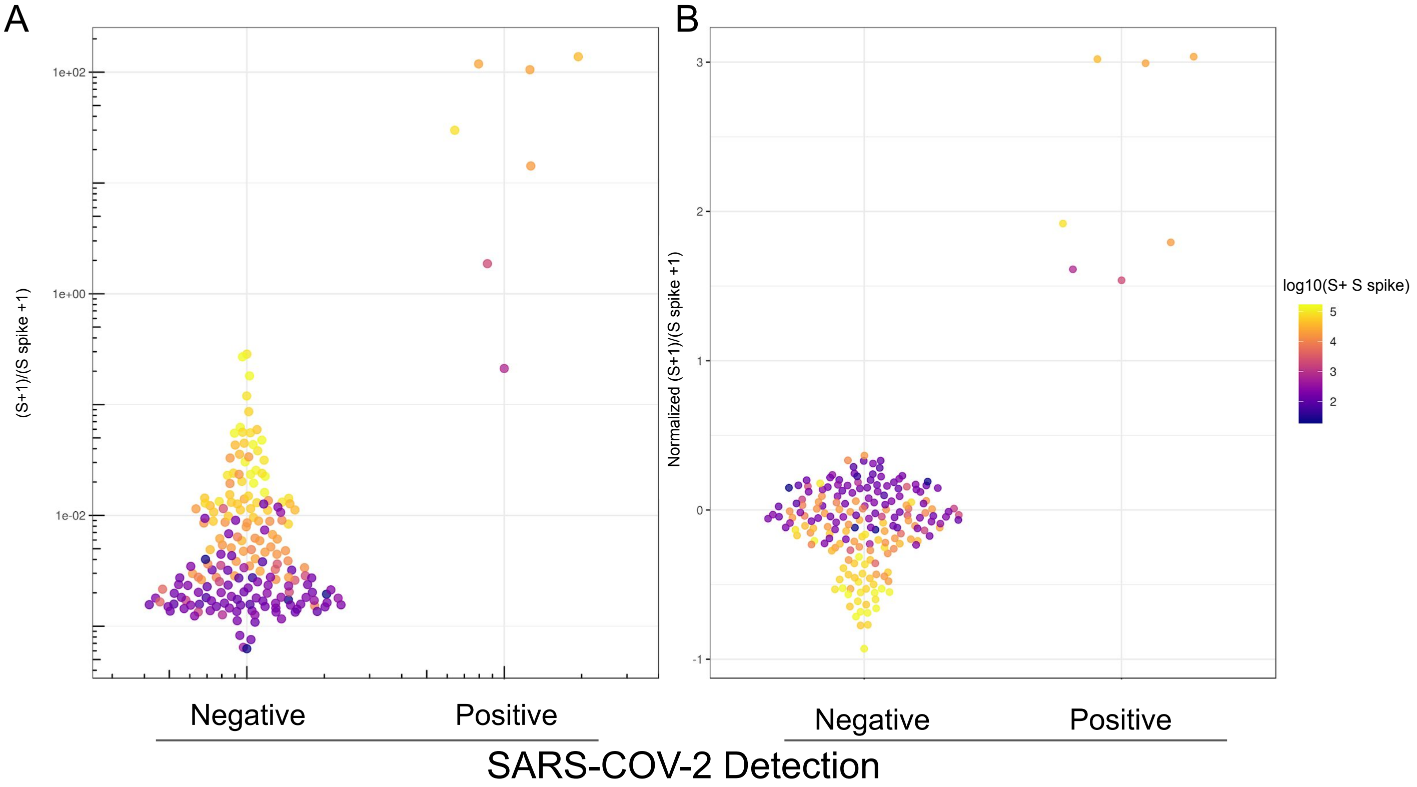

Supplementary Fig. 20. Computational correction for index mis-assignment using a mixed-model. To expand the number of samples we are capable of testing, we can use a combinatorial indexing strategy. In this experiment we used a single index on i5 to uniquely identify a plate and 96 i7 indices to identify wells. (A) The ratio of S to S spike (y-axis) is plotted for clinical samples based on whether Covid was detected by RT-qPCR (x-axis). SARS-CoV-2 positive samples were filtered to have Ct<32. The effects of index mis-assignment across plates can be observed as i7 indices that have high a sum of S and S spike across all samples that share the same i7 barcode across plates (colors). (B) Best linear unbiased predictor residuals are plotted (y-axis) for data in A, after computational correction of the log10(S+1/S spike+1) ratio by treating the identity of the i7 barcode as a random effect.

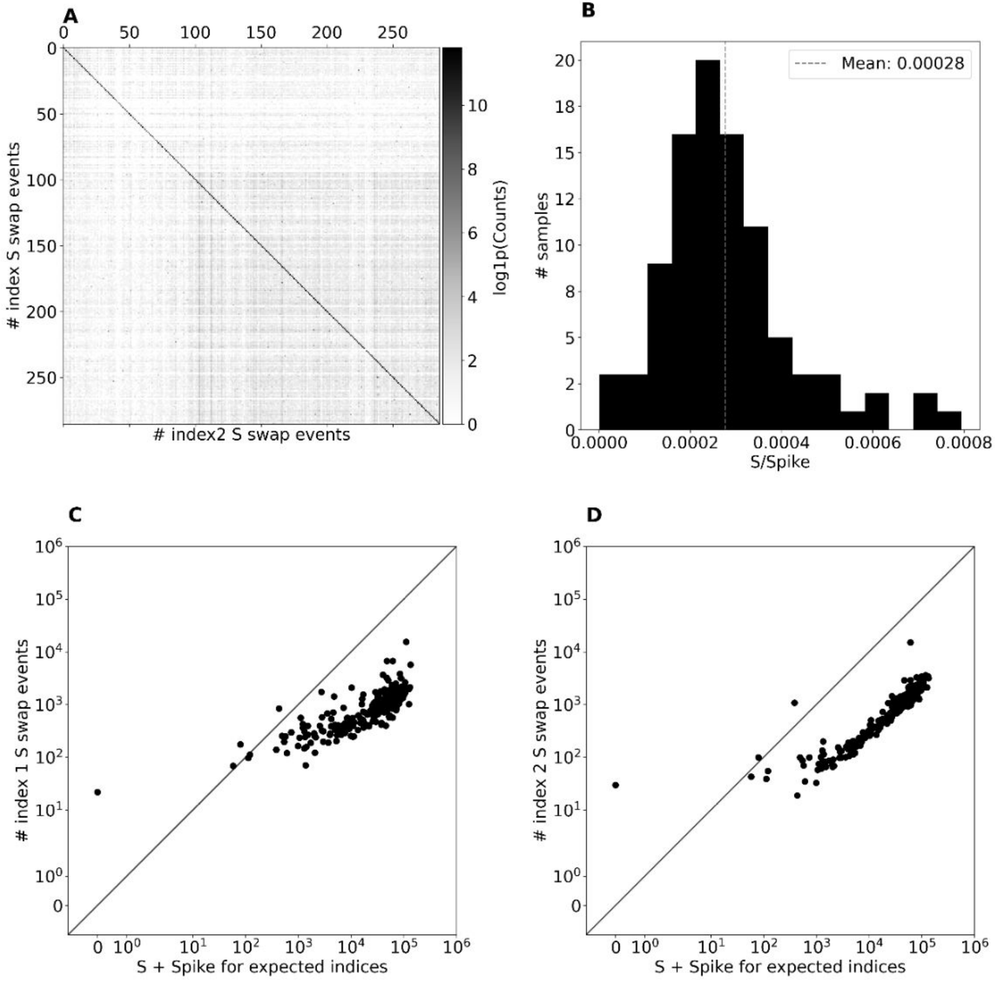

Supplementary Fig. 21. Quantifying the role of index mis-assignment as a source of noise in the S reads. A) A matching matrix for the viral S + S spike count for each pair of i5 and i7 index pairs from run v19 that used a unique dual index design. The index pairs along the diagonal correspond to expected index pairs for samples present in the experiment (expected matching indices) and the index pairs off of the diagonal correspond to index mis-assignment events. B) The distribution of ratios of viral S counts to Spike counts for samples with known zero amount of viral RNA. The mean ratio is 0.00028. C) The number of i7 mis-assignment events vs the number of viral S + S Spike counts for each sample. D) The number of i5 mis-assignment events vs the number of viral S + S Spike counts for each sample.

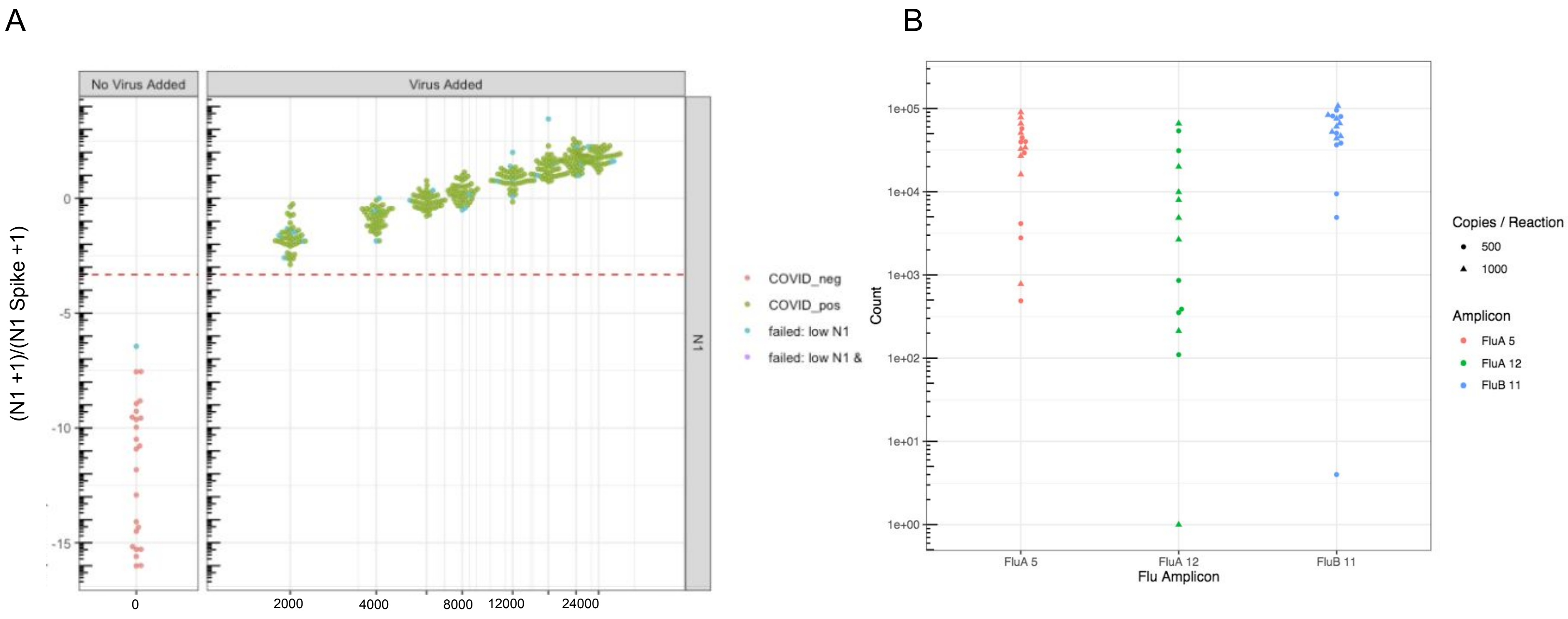

Supplementary Fig. 22. Extension of the SwabSeq Assay. A) We developed and tested to multiplex with additional SARS-CoV-2 amplicons. Here we demonstrate the N1 Amplicon LOD in saliva is around 2000 copies per mL. B) Testing of the three flu amplicons demonstrates that we are able to detect by sequencing samples with 500-1000 copies / reaction in PBS.

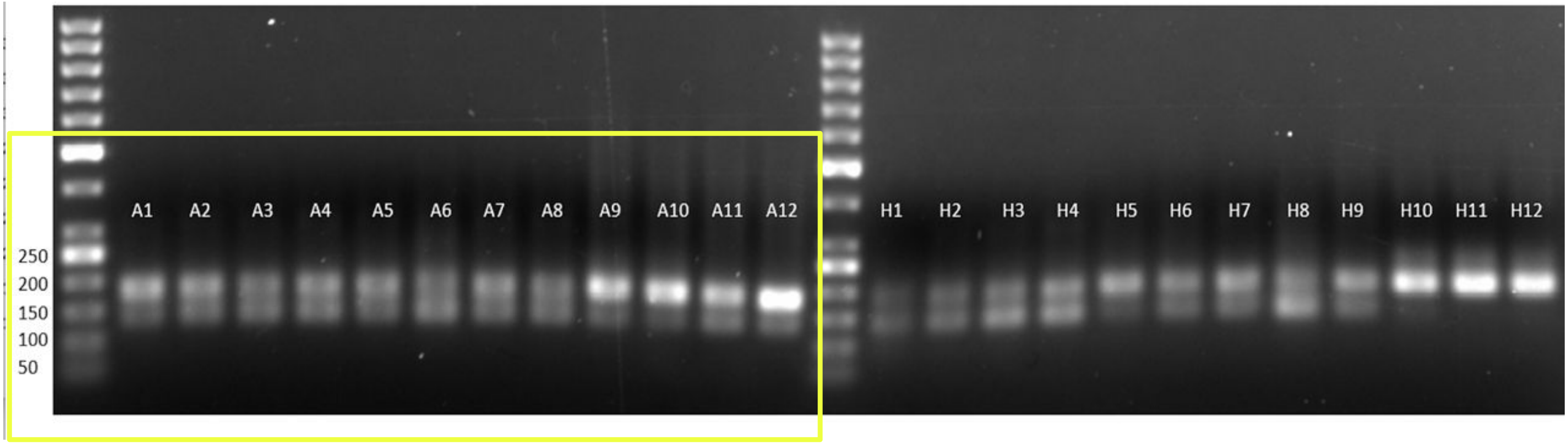

Source Data for Supplementary Fig. 14. Uncropped gel. Cropped Region for Supplementary Figure 14 is shown in yellow.

Table of Contents

1. PURPOSE 2
2. MATERIALS 2
2.1. Reagents and equipment 3
2.2. Primers 3
3. METHOD 4
3.1. Preparing Primer Plates 4
3.2. Construction and dilution of *in vitro* S RNA standard 5
3.3. *In vitro* S standard dilution protocol 6
3.4. Using the Diversified S RNA Standard 7
3.5. Samples testing on the SwabSeq Platform. 8
3.5.1. RNA-purified samples, Thermofisher Kingfisher platform 8
3.5.2. Saliva Specimen Processing 9
3.5.3. Extraction Free Nasal Swab processing 9
3.6. RT-PCR 9
3.6.1. *Make Master Mix:* 9
3.6.2. *Tracking sample plate (refer to sample set up sheet)* 10
3.6.3. *Putting Together PCR Plate* 10
3.6.4. *Reverse Transcription and PCR* 11
3.7. POST-PCR PROCESSING 11
3.7.1. *PCR products Combined and purified.* 11
3.7.2. *Bead Cleanup* 11
3.7.3. *Library Quantification and Quality Control using Traditional S RNA Standard* 11
3.7.4. *Library Quantification and Quality Control using Diversified S RNA Standard* 12
3.8. SEQUENCING PRIMER MIXES AND LOADING 12
3.8.1. *MiniSeq Primer Mixes* 12
3.8.3. *NextSeq Primer Mixes* 12
3.8.4. *Next Seq Sequencing* 13
4. DATA ANALYSIS 13
4.1. *MiSeq Control Software:* 13
4.2. Real Time Analysis Software: 13
4.3. bcl2fastq Conversion Software: 13
4.4. UCLA Sample Demultiplexing and Amplicon Counting Software: 13
5. RESULT INTERPRETATION FOR PURIFIED SAMPLES 14

6. **RESULT INTERPRETATION FOR EXTRACTION-FREE SALIVA AND NASAL SWAB** 15

1. PURPOSE

SwabSeq technology presents a unique approach to viral testing that eliminates many of the current bottlenecks that limit clinical testing and importantly can rapidly scale to 10,000 samples per day with simple automation and many of the machines and semi-automation that are standard in research laboratories. In this operating procedure, we provide the list of reagents and protocols and in addition we outline the methods for using SwabSeq with alternative specimen types such as extraction free saliva and extraction free nasal swabs.

2. MATERIALS

NOTE: Do not use any reagent beyond the expiration date

Table 1. List of reagents.

**Reagent**	**Storage Temperature**	**Preparation Details (note here or refer to procedure)**
S Standard	-80°C	See Section C, page 6
Primers	-20°C	See Section B, page 3
magMAX Viral/Pathogen Nucleic Acid Isolation Kit	4°C-8°C and RT	See Section D, Page 9
TBE Buffer (Tris-borate-EDTA) (10X)	15°C -30°C	See Section E, page 10
Nuclease Free Water	15°C -30°C	See Section F, page 10
Tween-20	15°C -30°C	See Section E, page 22
TaqPath™ 1-Step RT-qPCR Master Mix (Fisher Scientific, A15300)	-20°C	See Section G, page 12
Ampure XP Beads (Fisher Scientific, A63880)	4°C-8°C	See Section H, page 14
Qubit™ RNA HS Assay Kit (ThermoFisher, Q32855)	4°C-8°C and RT	See Section H, page 14
PhiX Control v3 (Illumina, FC-110-3001)	4°C-8°C and RT	See Section H, page 14
MiSeq Reagent Kit v3 (150-cycle, Catalog # MS-102-3001)	-20°C	See Section I, page 15
NextSeq 500/550 High Output Kit v2 (150 cycles, #20024907)	-20°C	See Section I, page 15

Table 2. Acceptable specimen, storage and handling.

**Acceptable Specimens**	**Storage Temperature**	**Storage Time**
Nasal Swabs	4°C, -20°C	72 hours, 1 week
Saliva	4°C, -20°C	72 hours, 1 week

2.1. Reagents and equipment

General
Capit-All (Thermofisher Scientific)
IntelliXcap Capper Decapper (Brooks)
Pipette Tips LQR LTS 20µL FL 960/10 (Rainin, 17014400 for Bench Smart)
12.5 µl GRIPTIP Sterile, Filter, LONG, 5 XYZ Racks of 384 Tips, Low Retention (Integra , 6505)
384-WELL HARDSHELL PLATE CLEAR (#20 PCS PK 4483285 Fisher)
TaqPath™ 1-Step RT-qPCR Master Mix (Fisher Scientific, A15300)
TE Buffer, Tris-EDTA, 1X Solution, pH 7.4 (Fisher BP24761)
Tween-20
EtOH
NUCLEASE-FREE WATER (Thermofisher, AM9937)
96-well plates (qRT-PCR)
Normal Saline
10X TBE 1M Tris-HCl pH8.0, 900mM boric acid, and 10mM EDTA(10x Buffer, ThermoFisher)

Synthetic S Standard Construction
HiScribe™ T7 High Yield RNA Synthesis Kit (New England Biolabs, E2040S)
DNAse, RNAse Free (NEB, M0303S)
RNA Clean & Concentrator-5 (Zymo, R1013)

Post-PCR Pooling and Purification
DynaMag-2 Magnet (Thermofisher Scientific, 12321D)
Ampure XP Beads (Fisher Scientific, A63880)
1.7 mL Eppendorf Lo-Bind Tubes (Fisher Scientific, 13-698-791)
150 mL reservoir, sterile, bulk, automation friendly, polystyrene (Integra, 6318)
Qubit™ RNA HS Assay Kit (ThermoFisher, Q32855)
Qubit™ Assay Tubes (Thermofisher Scientific, Q32856)
Qubit™ RNA BR Assay Kit (ThermoFisher, Q10211)

Sequencing Materials and Reagents
PhiX Control v3 (Illumina, FC-110-3001)
Illumina Free Adapter Blocking Reagent (48 reactions, # 20024145)
MiSeq Reagent Kit v3 (150-cycle, Catalog # MS-102-3001)
NextSeq 500/550 High Output Kit v2 (150 cycles, #20024907)

2.2. Primers

Indexed Primers
Custom Primer sets were designed to amplify the S gene in the SARS-CoV2 genome and the RPP30 gene in the human genome. The S gene amplification indicates the presence of SARS-CoV2 RNA genome within the specimen. The RPP30 gene demonstrates adequate sample collection. Each primer pair (i5 and i7, designated as F and R) has an 10-basepair random barcode adapted to the primer that we have designed to be unique to each i7 barcode which represents the plate-well for that barcode. The i5 barcode can be repeated over the plate and is sometimes referred to as the "plate barcode". The combinatorial indexing strategy limits

the number of unique primers that need to be purchased. An alternative, but more expensive strategy, would be to purchase unique-dual indices (UDI) which are designed to be a unique pair in each well.

S and RPP30 Primers, Custom Primer Design is attached as a separate document "UCLA_SwabSeq_barcodedPrimers_V4"
Ordered from IDT
100nM, 200uM concentration, Standard Purification

Final Concentration for Working Primers (10x):
4 µM for S
0.5 µM for RPP30

Custom Sequencing Primers
These primers are for custom sequencing approaches on the Illumina based flow cell.
Ordered from Integrated DNA Technologies
100nM synthesis scale, concentration is 100uM

Table 3. Primers used for specific amplification of S gene and human control RPP30.

**S_SARS-CoV-2**		**Tm**
Read_1	gctggtgctgcagcttattatgtgggt	63
i7_seq	agatgctgtagactgtgcacttgaccct	63
i5_seq	acccacataataagctgcagcaccagc	63
**RPP30**		
Read_1	gagcggctgtctccacaagtccg	63.5
i7_seq	acccgctcgcaggtccaaatct	62.6
i5_seq	cggacttgtggagacagccgctc	63.5

3. METHOD

3.1. <u>Preparing Primer Plates</u>

All plates will be pre-stamped with a mix of indexed primers that are 10x concentration (4 µM for SFor and SRev; 0.5 µM for RPP30For and RPP30Rev). For a 20 µL reaction, we will place 2uL into each well of the primer plate. These were ordered in bulk from IDT.

1) Spin down master plates at 2000xg for 1 minute to ensure that all frozen ice is at the bottom of the well. This prevents cross contamination of indexed primers when removing the foil lid.
2) Set up Integra work station:
    a) Scan or write down Barcode Labels in the Primer Plate Notebook

b) Label multiple 384-well primer plates with primer set name.
c) Change the Setting on the Integra for multi-dispensing of 2uLs per plate x 6 plates

3) Remove seal from the master plates very carefully
4) Using the 384-well head for the Integra Viaflo, carefully pipet up primer plates and dispense 2uL into each plate.
5) Seal each plate, freeze in -20 until use.

Repeat with each 384-well Primer Plate Set.

An alternative approach is to purchase pre-stamped primer plates at the needed concentrations directly from the company synthesizing the plates. We have explored these options with a number of oligo synthesizing companies and have found that although it requires validation and resources for set up, it can drastically reduce the labor required to accurately pipet the primer plates.

3.2. Construction and dilution of *in vitro* S RNA standard

Purpose: We use an *in vitro* S RNA standard as a control in each of our wells. The sequence is meant to mimic the actual SARS CoV-2 amplicon (same amplicon structure except for a 6 nt unique stretch to distinguish). The use of this synthetic standard has two important advantages. This can be done in large batches every 6-months to 1-year or as needed depending on the rate of usage.

For this in vitro standard, sequencing requires the addition of high percentage of PhiX in order to assist with cluster generation. To simplify our process, we currently use the diversified standard (section 3.4) that was pre-synthesized for our current testing needs.

1. This serves as an in-well positive control for the S primers. Even in a sample that has no SARS-CoV-2, we can observe amplification with primers, thereby ensuring that a negative result is due to lack of virus, and not due to technical issues with the primers.
2. A second benefit is that we can use the ratio between S/S standard in our analysis pipeline. This has the effect of demonstrating that small changes in experimental conditions due to differences in samples, noise and amplification biases are not driving our results.

RT-PCR primers for standard constriction using *In vitro* Transcription (adding on T7 promoter)

Table 4. Primers to construct template for the S Synthetic standard.

S_FP	TAATACGACTCACTATAGggctggtgctgcagcttattatgtgggtATAGAAcaacctaggacttttctattaa
S_RP	aacgtacactttgtttctgagagagg

1. Perform RT-PCR in a 96-Well thermocycler using the primers above and gRNA of SARS-CoV-2.
2. Run on an Agarose gel and ensure specific products at ~130 bp
3. Purify DNA using Ampure Beads, use a ratio of 1.8 ratio of beads: sample volume.
4. Vortex and let sit for 5 minutes at room temperature.
5. Use magnet to collect beads for 1 minute.
6. Remove liquid and wash beads twice with 500 ul of freshly made 70% EtOH.
7. Elute in 100uL of 0.1X Qiagen EB buffer.
8. Use a magnet to collect beads for 1 minute. Transfer 90 uL to new eppendorf tube.
9. Quantify samples using Qubit DNA BR Kit.

Store DNA overnight or at -20 for long term storage if not immediately proceeding to IVT reaction.

10. Thaw the necessary kit components from the NEB HiScribe Kit. Mix and pulse-spin in microfuge to collect solutions to bottom of tubes. Keep on ice.
11. Prepare MasterMix for In Vitro Translation Reaction:
    a. If you are planning to run many reactions, it is convenient to prepare a master mix by combining equal volumes of the 10X reaction buffer and four ribonucleotide (NTP) solutions
    b. Using this mastermix (see below Table 1) we made a 4 reactions.
    c. Split into 4 tubes with 20 µl each.
    d. Vortex, pulse spin, but at 37˚C overnight in Thermocycler machine.

Table 5. MasterMix for IVT to make Synthetic Standard S

**Component**	**per reaction**	**Property**	**4 reactions (µl)**
Nuclease-free Water	2.5 µl		10
10X Reaction Buffer	1.5 µl	0.75X final	6
NTP	1.5 µl each	7.5 mM each final (4µL total)	6
Template DNA	13 µl	~300-600ng template	52
T7 RNA Polymerase Mix	1.5 µl		6
Total reaction volume	20 µl		80

Post-IVT Purification

1. After IVT, we DNAse treated the reactions by adding 1uL of DNase (NEB, M0303S) to each reaction
2. Incubate reactions at 37°C for 10 minutes.
3. Add 1 µl of 0.5 M EDTA (to a final concentration of 5 mM).
4. Heat inactivate at 75°C for 10 minutes.
5. RNA is purified using the Zymo RNA clean and concentrator column.
6. RNA is quantified with an Agilent TapeStation or Qubit using the RNA BR Kit.

3.3. *In vitro* S standard dilution protocol

The Synthetic S standard is an internal well control for the S primer pair. This standard is placed directly in the master mix and diluted to the same copy number in every well. Therefore, even for samples in which there is no SARS-CoV-2 virus present, we have an internal control demonstrating that the reaction conditions were sufficient for amplification with the S primer pair. The key for the synthetic S standard is to include it at a concentration of 100-1000 copies per reaction. Therefore, quantitation of the synthetic S standard is key to this experiment; if the quantitation is off by an order of magnitude, the synthetic standard can overwhelm signal seen in the experiment. Our current working protocol uses 250 copies per reaction.

Due to the sensitivity of the standard dilution quantitation, we suggest making small aliquots that are thawed per run every month and performing RT-qPCR to quantify the copies/reaction. Aliquots are then created from this batch and stored at -80ºC to minimize freeze thaws.

Protocol for Creating Standard Dilutions:
This should be done fresh every month to prevent degradation of the standard dilutions. Each month, enough aliquots should be created to last the estimated number of runs for the month.

1) Clean Biosafety Hood with 10% bleach and treat bench with UV light for 15 minutes
2) Treat pipettes, 0.1% Tween-20 in water with UV light for 15 minutes
3) Dilute and aliquot standard dilution into single use tubes:
    a) Measure the concentration of the S stock using HS RNA Qubit.
    b) Using the concentration from the previous step, calculate copies/uL using the equation found using the NEB Bio Calculator (nebiocalculator.neb.com). RNA length is 130 nt.
    c) Perform serial dilutions of 100-fold each using 0.1% Tween-20 in water until a concentration of ~10,000 copies/uL is reached. Save an aliquot of each dilution for quantification in step 5.
    d) Aliquot the final dilution into individual tubes containing 10uL each. These will be used to standard the master mix for each run of Swabseq.
4) *Perform a Qubit analysis on the first 100-fold dilution to validate.*
5) QC via RT-qPCR and validate copies/uL. Ct for 100 copies should come up between 33 and 34; for 1000 copies should come up around Ct of 30.

3.4. Using the Diversified S RNA Standard

Purpose: Next-Generation Sequencing of a small number of amplicons requires the addition of PhiX standard to the library to provide sufficient diversity for cluster generation and short-read sequencing. We have developed a diversified version of the S RNA standard that provides sufficient library diversity without the need for PhiX. These can be purchased from an Synthego.

To prepare the diversified S RNA standard, prepare the 4 separate diversified S RNA standards as above. Combine equimolar concentrations of the 4 standards. Dilute according to the dilution protocol outlined above (Section 3.3).

Table 6: Diversified S RNA Standard Sequences

S2_001	GCTGGTGCTGCAGCTTATTATGTGGGTGTGTATCTCACGAAGCGACCCTTTGGAAAATATAATGAAAATGGAACCATTACAGATGCTGTAGACTGTGCACTTGACCCT
S2_002	GCTGGTGCTGCAGCTTATTATGTGGGTCCTCGCTAGGACGTCGCTATgacgccAAAATATAATGAAAATGGAACCATTACAGATGCTGTAGACTGTGCACTTGACCCT
S2_003	GCTGGTGCTGCAGCTTATTATGTGGGTAGCACGACTTGATCTAACTgacactaAAAATATAATGAAAATGGAACCATTACAGATGCTGTAGACTGTGCACTTGACCCT
S2_004	GCTGGTGCTGCAGCTTATTATGTGGGTTAAGTAGGACTTCGATTggaTggaatAAAATATAATGAAAATGGAACCATTACAGATGCTGTAGACTGTGCACTTGACCCT

3.5. Samples testing on the SwabSeq Platform.

Swabseq can take in a variety of sample types. It performs very well with purified samples, however these are not as conducive to scale due to the labor-intensive RNA-purification process (Section 3.5.1). We have optimized our process with extraction free protocols for both nasal swabs into TE buffer (Section 3.5.2) and for saliva (Section 3.5.3)

3.5.1. RNA-purified samples, Thermofisher Kingfisher platform

Nasal Swabs collected from into any of the traditional collection buffers (e.g., Aimes Buffer, Normal Saline or VTM) can be used for this protocol. Set up 96-well deep well plate for 94 samples. We use the same volumes, regardless of the type of collection media that is used. This process is performed at the UCLA Clinical Microbiology Laboratory in Brentwood Annex.

1) Fill plates with following liquids
    a. Wash Plate 1: 1mL Wash Buffer
    b. Wash Plate 2: 1mL 80% EtOH
    c. Elution Buffer Plate: 100 uL per well
2) Prepare Bead Binding Mix
    a. Prepare required amount on each day of use
    b. Vortex the total Nucleic Acid Magnetic Beads to ensure that the bead mixture is homogeneous
    c. Prepare the bead binding mix according to the table below

Component	Volume per well[1]
Binding Solution	530 µL
Total Nucleic Acid Magnetic Beads	20 µL
**Total volume per well**	550 µL

[1] Include 10% overage when making the Binding Bead Mix for use with multiple reactions.

    d. Mix well by inversion, store are room temperature until use
3) Digest with Proteinase K
    a. Add 1-uL of Proteinase K into each well of the Kingfisher Deepwell 96 plate
    b. Pipet 400uL of each patient sample into a 96-well format, leave PPC and NPC control well empty
    c. Add 400uL of Nuclease Free water to the negative control well
    d. Invert Binding Bead Mix 5 times gently to mix, then add 550 uL to each sample and negative control well
4) Seal the plate with the MicroAmp Clear Adhesive Film.
5) Load the King Fisher Flex Machine:

Plate ID	Plate position	Plate Type	Reagent	Volume Per Well
Wash 1 Plate	2	KingFisher Deepwell 96 Plate	Wash Buffer	1,000uL
Wash 2 Plate	3		80% Ethanol	1,000uL
Elution Plate	4		Elution Solution	100uL
Tip Comb Plate	5	Place a KingFisher 996 Tip comp for DW magnets in a King Fisher 96 KF microplate		

6) Select the MVP_2Wash_400_Flex on the KingFisher Flex Magnetic Particle Processor with the 96 Deep Well Head.
7) Start the run and load the prepared plates into position when prompted by the instrument

8) Remove the elution plate after the run is complete (~24 minutes later) and cover with Clear Adhesive Film.
9) Place elution plate on ice for use in RT-PCR until ready to load sample.
10) Pipet 7uL from deepwell sample plate into designated quadrant of 384 well plate using the Rainin Benchsmart 200uL.

3.5.2. Saliva Specimen Processing

Saliva samples will be processed using extraction free methods. All samples will be received in the UCLA COVID19 testing lab in 650 Charles E Young Dr South, CHS Building for direct accessioning and processing.

1) Fresh Saliva is collected into a pre-barcoded tube by the user through a plastic funnel. All samples are stored and transported at 4°C.
2) Tubes with samples are placed in 96 well racks in a 95°C circulating water bath for 30 minutes.
3) Tubes are spun in 4°C centrifuge for 20 seconds at 200xg to collect saliva at bottom of tube.
4) Rack is placed in the automated decapper (Brooks Intellecap or ThermoFisher Capit-All) and the 96 tubes are uncapped.
5) Using the Benchsmart 200, we remove the topmost 200uL from the tubes at high speed to prevent overflow. Aspirate 40uL of saliva from just below the bottom of the tube and dispense back into the tube in increments of 5uL until there are no air bubbles at the bottoms of the tips. Dispense 5uL into the appropriate quadrant of the 384-well plate pre-loaded with primers and mastermix.
6) After each quadrant is dispensed into the 384-well plate, the 384 well plate is placed into the thermocycler and run with the RT-PCR reaction described below.

3.5.3. Extraction Free Nasal Swab processing

Extraction Free Nasal Swab Specimens are NP, Mid-Nasal and Anterior Nares samples collected directly into 750uL of Tris-EDTA (TE). All nasal swabs collected in this manner will be received in the UCLA COVID19 testing lab in 650 Charles E Young Dr South, CHS Building for direct accessioning and processing.

1) Nasal Swabs collected into pre filled tubes with 750uL TE are stored and transported at 4°C.
2) Tubes are placed into a 96 well rack and placed in a 95°C circulating water bath for 30 minutes.
3) Samples are spun in 4°C centrifuge for 20 seconds at 200xg to collect nasal swab sample at bottom of tube.
4) Rack is placed in the automated decapper (Brooks Intellecap or ThermoFisher Capit-All) and the 96 wells are uncapped.
5) Using the Benchsmart 20, we pipet 2.5 uL of Nuclease Free water, pipet an airgap of 2.5 uL, and then pipet 2.5uL of Extraction Free Nasal Swab. The total liquid volume of 5uL is dispensed into a designated quadrant of a 384-well plate pre-loaded with primer and mastermix.
6) After each quadrant is dispensed into the 384-well plate, the 384 well plate is placed into the thermocycler and run with the RT-PCR reaction described below.

3.6. RT-PCR

3.6.1. *Make Master Mix:*

(This calculation is for a single 384 well plates). This process is the same regardless of the sample type above (purified RNA from nasal swab, extraction-free nasal swab, extraction-free saliva)

Reagents:
TaqPath™ 1-Step RT-qPCR Master Mix (Fisher Scientific, A15300)
NUCLEASE-FREE WATER (Thermofisher, AM9937)
S Standard Dilution (see section G)

Table 7. MasterMix calculation.

**Mastermix Calculation**			
**per 384 well plate**			
	**RT-PCR mix:**	**uL or copies per reaction**	**Total**
	4x Mastermix	5 uL	1920
	Water	8 uL	3702
Dilution 4	S RNA Standard quant 250 copies *384	192,000 copies	Calculate from dilution
Sample	5		
indexed primers	2		
Total Volume			20
Total Mastermix per well	13		

Notes:
- Master mix is made in a clean hood that has been treated for 15 minutes with UV light and cleaned with 10% bleach solution.
- All pipettes and water used are also treated with UV light

3.6.2. *Tracking sample plate (refer to sample set up sheet)*

1) Each plate will be designated a quadrant: 1, 2, 3 or 4 of the 384-well plate. Record the Barcode and quadrant for each 96-well plate or tube-rack of samples.

3.6.3. *Putting Together PCR Plate*

1) Clean benchtop surface with 10% Bleach
2) RNAse inhibitor treatment of benchtop surface
3) Take out pre-stamped primer plates (see section D) from -20 freezer and thaw on ice
4) Spin plates down at 2000xg for 1 minute
5) Visually inspect to ensure there is primer in each well
6) Pipet 13 uL of master-mix into each well using the Rainin Benchsmart 20 or PrePCR Integra Viaflo 12.5uL

7) Add 5ul of sample as described above in sections D, E and F for purified RNA from nasal swabs, extraction free saliva or extraction-free nasal swabs.

3.6.4. *Reverse Transcription and PCR*

1. Program the following into the 384 Well Veriti Thermocycler.

Table 8. PCR cycle.

1	55C	10 min
2	95C	1 min
3	95C	10 sec
4	60C	30 sec
	Go to step 3, 40 cycles (purified samples) or 50 cycles (unpurified samples)	
	Hold at 12C	

2. Load plate into 384 well cycler
3. Press Start

3.7. <u>POST-PCR PROCESSING</u>

3.7.1. *PCR products Combined and purified.*

1) Use Integra Viaflow, pipet 6 ul from each well of a 384 well plate into a sterile reservoir.
2) Repeat step one for each 384 well plate that will be combined into a sequencing reaction run. All samples will be combined into the same reservoir.
3) Slightly tilt the reservoir back and forth to mix.
4) Transfer the entire volume to a 15 mL conical tube and vortex thoroughly.
5) Transfer 100 ul to an eppendorf tube.

3.7.2. *Bead Cleanup*

1) Add 50 ul of AmpureXP beads (0.5:1 ratio of beads: sample volume) to 100 ul volume of pooled PCR reaction. Vortex and let sit for 5 minutes at room temperature.
2) Use magnet to collect beads for 1 minute.
3) Transfer supernatant (~150 ul) to a new eppendorf tube.
4) Add 130 ul of AmpureXP beads to the 150 ul of supernatant. Vortex and let sit for 5 minutes at room temperature.
5) Use magnet to collect beads for 1 minute.
6) Remove liquid and wash beads twice with 500 ul of freshly made 70% EtOH.
7) Elute in 40 ul of qiagen EB buffer
8) Use a magnet to collect beads for 1 minute. Transfer 33 ul to new eppendorf tube.

3.7.3. *Library Quantification and Quality Control using Traditional S RNA Standard*

Note: follow either this protocol or the one listed in the next section depending on which type of S RNA standard you plan to use (either traditional or diversified)

1) Make a 1:10 dilution of eluted library using Ultrapure Water.
2) Use High Sensitivity DNA Qubit to measure the concentration of the 1:10 dilution.

3) Use the following link to determine the concentration of the 1:10 dilution in nM - https://support.illumina.com/bulletins/2016/11/converting-ngl-to-nm-when-calculating-dsdna-library-concentration-.html. Use 195bp for size.
4) Based on the above calculation, make a 5 nM dilution from the 1:10 dilution.
5) Measure this 5nM dilution and a stock of Illumina PhiX using High Sensitivity DNA Qubit.
6) Use the link from step 3 to calculate the concentration in nM of the 5nM dilution and the Illumina PhiX stock.
7) Make a dilution of the PhiX stock to equal the nM concentration of the 5nM dilution. For example, if the"5nM" dilution was actually 4.39 nM, dilute PhiX down to 4.39 nM.
8) Combine 14 ul of the "5 nM" dilution with 5ul of the dilution of PhiX. This results in a "5nM" library (4.39 nM in the example) that is 30% PhiX and 70% the library of interest.
9) Run the above 5nM library on an Agilent Technologies D1000 High Sensitivity Screentape.

3.7.4. *Library Quantification and Quality Control using Diversified S RNA Standard*

Note: follow either this protocol or the one listed in the next section depending on which type of S RNA standard you plan to use (either traditional or diversified)

1) Make a 1:10 dilution of eluted library using Ultrapure Water.
2) Use High Sensitivity DNA Qubit to measure the concentration of the 1:10 dilution.
3) Use the following link to determine the concentration of the 1:10 dilution in nM - https://support.illumina.com/bulletins/2016/11/converting-ngl-to-nm-when-calculating-dsdna-library-concentration-.html. Use 195bp for size.
4) Based on the above calculation, make a 5 nM dilution from the 1:10 dilution.
5) Measure the concentration of the 5nM dilution using the High Sensitivity DNA Qubit
6) Use the link above to calculate the concentration in nM of the 5nM dilution. ***Note: with the diversified S RNA standard, do NOT quantify and add PhiX to the 5nM dilution. Proceed to the next section.***

3.8. SEQUENCING PRIMER MIXES AND LOADING

3.8.1. *MiniSeq Primer Mixes*

a. Add 80 ul of water, 10 ul of S read 1 primer (100 uM stock), and 10 ul of RPP3 read 1 primer (100 uM stock) to an eppendorf tube labeled "Read 1 primer mix". Final concentration will be 20 uM of primers (10 uM of each read 1 primer).
b. Add 80 ul of water, 10 ul of S i7 primer (100 uM stock), and 10 ul of RPP3 i7 primer (100 uM stock) to an eppendorf tube labeled "i7 primer mix". Final concentration will be 20 uM of primers (10 uM of each i7 primer).

3.8.2. *MiniSeq Sequencing*

a. Load 24.5uL of the read 1 primer mix into reservoir 24. Mix.
b. Load 26uL of the i7 primer mix into reservoir 28. Mix.
c. Load 26uL of the i5 primer mix into reservoir 28. Mix.
d. Load 500uL 1.5pM library into reservoir 16.

3.8.3. *NextSeq Primer Mixes*

a. Add 80 ul of water, 10 ul of S read 1 primer (100 uM stock), and 10 ul of RPP3 read 1 primer (100 uM stock) to an eppendorf tube labeled "Read 1 primer mix". Final concentration will be 20 uM of primers (10 uM of each read 1 primer).
b. In addition to the read 1 primer and i7 primer mix above, the NextSeq requires an i5 primer mix. To make this, add 80 ul of water, 10 ul of S i5 primer (100 uM stock), and 10

ul of RPP3 i5 primer (100 uM) stock to an eppendorf tube labeled "i5 primer mix". Final concentration will be 20 uM of primers (10 uM of each i5 primer).

3.8.4. *Next Seq Sequencing*

a. Load 52uL of the read 1 primer mix into reservoir 20. Mix.
b. Load 85uL of the i7 primer mix into reservoir 22. Mix.
c. Load 85uL of the i5 primer mix into reservoir 22. Mix.
d. Load 1300uL 1.25 pM library into reservoir 10.

4. DATA ANALYSIS

Illumina BCL files are downloaded and converted into FASTQ sequencing files using Illumina's bcl2fastq software. Each amplicon sequence consists of a set of three individual reads: one 26 base pair read (read1) that identifies the amplicon (S, S standard, or RPP30) and two 10 base pair index reads (index1 and index2) that together uniquely identify the sample. Sequences are assigned to samples using the two index reads and the sum of the reads for each amplicon in each sample is obtained. Decisions about whether the sample passed QC and whether SAR-CoV-2 was detected in a sample are based on the count of sequences observed for each amplicon within each sample and explained in detail below.

4.1. *MiSeq Control Software:*

The MiSeq Control Software (Illumina Inc., 'For Research Use Only') controls the flow cell stage, temperature and fluidics system. It also captures images of clusters, generating image analysis, base calling, and base call quality data.

4.2. Real Time Analysis Software:

Primary analysis is performed by the Real Time Analysis (RTA) software (Illumina Inc., 'For Research Use Only') and consists of base calling of each cluster at each cycle. In addition to base calling, RTA assigns an analytical quality score (Q-score) to each base call. Calculations of Q-scores are based on the ratio of the signal intensity of the highest base in a given cluster during a given cycle to the signal intensity of the three other bases. The quality score Q is calculated as -10 log10 P, where P is the probability that base call is incorrect. A minimum of 80% of basecalls must meet the Q30 threshold to proceed to data analysis. If these criteria are not met this could be due to a lack of sequence diversity (insufficient PhiX concentration), a technical problem in library construction, a faulty flow cell or sequencing instrument failure. If fewer than 80% of basecalls meet the Q30 threshold the entire run is discarded.

4.3. bcl2fastq Conversion Software:

The bcl2fastq conversion software (Illumina Inc., 'For Research Use Only') is used to process BCL (base call log) files generated by the MiSeq instrument and convert time into FASTQ files. FASTQ is a standard text-based file format that will store the nucleotide sequences and base quality scores for each read sequenced from a sample. Three FASTQ files are generated, one corresponding to 26 base pairs of sequence within each amplicon (read1) and two 10 base pair index sequences (index1 and index2) that together uniquely identify each sample.

4.4. UCLA Sample Demultiplexing and Amplicon Counting Software:

Read1 is matched to one of the three expected amplicons allowing for the possibility of a single nucleotide error in the amplicon sequence. The set of three reads is discarded if read1 has a hamming distance greater

than 1 from the expected amplicons. Samples are demultiplexed using the two index reads. Demultiplexing means assigning the sequences to the sample from which they originated. Observed index reads are matched to the expected index sequences allowing for the possibility a single nucleotide error in one or both of the index sequences. The set of three reads are discarded if both index1 and index2 have hamming distances greater than 1 from the expected index sequences. The sum of reads for each amplicon and each sample is calculated.

A fully automated R package that runs our Amplicon Counting Software and generates quality-control reports and interprets results for patient samples can be found at: https://github.com/joshsbloom/swabseqr

The development version of scripts for the initial implementation of our analysis pipeline and Amplicon Counting Software can be found at https://github.com/joshsbloom/swabseq

5. RESULT INTERPRETATION FOR PURIFIED SAMPLES

We require that 10 reads are detected for RPP30 for each sample. This serves as a control that sample collection took place properly and contains a human specimen. If fewer than 10 reads are detected for RPP30 the results are considered inconclusive.

We require that the sum of S and S synthetic standard reads exceeds 2,000 reads or the results are considered inconclusive. The S synthetic standard is added to the master mix, is present in every well and every sample in our assay, and even if no virus is present, if the primers and the assay are working properly the S synthetic standard will amplify and be sequenced. We have observed that samples with very high viral concentrations will result in high S reads and low S synthetic standard reads, and samples with low viral concentrations will result in low S reads and high S synthetic standard reads. In both cases the sum of S and S standard should be high in any sample regardless of the presence of Sars-CoV-2. This follows from the fact that the same S primers have equal preference for the S and S synthetic standard and amplify both with equal efficiency.

Assuming a sample as greater than 10 RPP30 reads and that the sum of S and S synthetic standard reads exceeds 2,000, we determine if SARS-CoV-2 is detected in a sample by seeing if the ratio of S to S standard exceeds 0.003. (We note that we add 1 count to both S and S standard before calculating this ratio to facilitate plotting the results on a logarithmic scale.) If the ratio is greater than 0.003 we concluded that Sars-CoV-2 is detected for that sample and if it is less than or equal to 0.003 we conclude that Sars-CoV-2 is not detected.

Table 9. Result interpretation for purified samples

**Well-controls**		**Results**			
**Total S + S Standard**	**RPP30 read count**	**S/S Standard ratio**	**Result**	**Interpretation**	**Action**
>2000 reads	>10	> 0.003	SARS- CoV-2 Detected	Positive for SARS-CoV-2 for the Sample ID.	Report results to physician, patient, and appropriate public health authorities.
>2000 reads	>10	< 0.003	SARS- CoV-2 Not Detected	Negative for SARS-CoV-2 for the Sample ID.	Report results to physician, patient, and appropriate public health authorities.

<2000 reads	>10		-	Inconclusive	Invalid for the Sample ID.	Quality control for the Sample ID is FAIL. Repeat sample or Recollect sample
>2000 reads	< 10		-	Inconclusive	Invalid for the Sample ID.	Quality control for the Sample ID is FAIL. Repeat sample or Recollect sample
<2000 reads	< 10		-	Inconclusive	Invalid for the Sample ID.	Quality control for the Sample ID is FAIL. Repeat sample or Recollect sample.

6. RESULT INTERPRETATION FOR EXTRACTION-FREE SALIVA AND NASAL SWAB

We require that the sum of S and S synthetic standard reads exceeds 500 reads, or the results are considered inconclusive. With inhibitory lysates we have observed that this lower total is acceptable for maintaining sensitivity and specificity while limiting the number of tests that are considered inconclusive. If the sum of S and S synthetic standard reads exceeds 500, we determine if SARS-CoV-2 is detected in a sample by seeing if the ratio of S to S standard exceeds 0.0 5. (We note that we add 1 count to both S and S standard before calculating this ratio to facilitate plotting the results on a logarithmic scale.) If the ratio is greater than 0.05 we concluded that Sars-CoV-2 is detected for that sample and if it is less than or equal to 0.05 we conclude that Sars-CoV-2 is not detected as long as 10 reads are detected for RPP30 for that sample. This serves as a control that sample collection took place properly and contains a human specimen. If fewer than 10 reads are detected for RPP30 and the ratio of S to S standard is less than or equal to 0.05 the results are considered inconclusive. We have modified this criteria such that only samples without Sars-CoV-2 signal are considered inconclusive if RPP30 reads are fewer than 10. This ensures that we do not miss Sars-Co V-2 positive samples that may have fewer RPP30 reads.

Table 10. Result interpretation for extraction-free saliva and nasal swab samples

**Well-controls**			**Results**			
**Total S + S Standard**	**RPP30 read count**		**S/S Standard ratio**	**Result**	**Interpretation**	**Action**
>500 reads	>0		> 0.05	SARS- CoV-2 Detected	Positive for SARS-CoV-2 for the Sample ID.	Report results to physician, patient, and appropriate public health authorities.
>500 reads	>10		< 0.05	SARS- CoV-2 Not Detected	Presumptive Negative for SARS-CoV-2 for the Sample ID.	Report results to physician, patient, and appropriate public health authorities.
<500 reads	>0		-	Inconclusive	Invalid for the Sample ID.	Quality control for the Sample ID is FAIL. Repeat sample or Recollect sample

>500 reads	< 10		<.05	Inconclusive	Invalid for the Sample ID.	Quality control for the Sample ID is FAIL. Repeat sample or Recollect sample
